# Golgi pH elevation due to loss of V-ATPase subunit V0a2 function correlates with tissue-specific glycosylation changes and globozoospermia

**DOI:** 10.1007/s00018-024-05506-7

**Published:** 2024-12-16

**Authors:** Johannes Kopp, Denise Jahn, Guido Vogt, Anthi Psoma, Edoardo Ratto, Willy Morelle, Nina Stelzer, Ingrid Hausser, Anne Hoffmann, Miguel Rodriguez de los Santos, Leonard A. Koch, Björn Fischer-Zirnsak, Christian Thiel, Wilhelm Palm, David Meierhofer, Geert van den Bogaart, François Foulquier, Andreas Meinhardt, Uwe Kornak

**Affiliations:** 1 Charité –Universitätsmedizin Berlin, corporate member of Freie Universität Berlin and Humboldt-Universität Zu Berlin, Institute of Medical Genetics and Human Genetics, 13353 Berlin, Germany; 2 Max Planck Institute for Molecular Genetics, RG Development & Disease, 14195 Berlin, Germany; 3 Institute of Chemistry and Biochemistry, Department of Biology, Chemistry and Pharmacy, Freie Universität Berlin, 14195 Berlin, Germany; 4 Berlin Institute of Health at Charité-Universitätsmedizin Berlin, Julius Wolff Institute - Center for Musculoskeletal Biomechanics and Regeneration, 13353 Berlin, Germany; 5 Department of Molecular Immunology (MI), University of Groningen, 9747AG Groningen, The Netherlands; 6 Cell Signaling and Metabolism, German Cancer Research Center (DKFZ), 69120 Heidelberg, Germany; 7 Faculty of Biosciences, University of Heidelberg, 69120 Heidelberg, Germany; 8 University of Lille, CNRS, UMR 8576 - UGSF - Unité de Glycobiologie Structurale et Fonctionnelle, 59000 Lille, France; 9 Institute of Pathology, Heidelberg University Hospital, 69120 Heidelberg, Germany; 10 Icahn School of Medicine at Mount Sinai, New York, NY 10029 USA; 11 Centre for Child and Adolescent Medicine, Department I, University Hospital Heidelberg, 69115 Heidelberg, Germany; 12 Max Planck Institute for Molecular Genetics, Mass-Spectrometry Facility, 14195 Berlin, Germany; 13 Institute of Anatomy and Cell Biology, Justus-Liebig-Universität Gießen, 35385 Gießen, Germany; 14 Institute of Human Genetics, University Medical Center Göttingen, 37073 Göttingen, Germany

**Keywords:** V-ATPase, Golgi, pH-regulation, Vesicular trafficking, Glycosylation, Cutis laxa, Neuronal migration, Spermiogenesis, Globozoospermia

## Abstract

Loss-of-function variants in *ATP6V0A2*, encoding the trans Golgi V-ATPase subunit V0a2, cause wrinkly skin syndrome (WSS), a connective tissue disorder with glycosylation defects and aberrant cortical neuron migration. We used knock-out (*Atp6v0a2*^−/−^) and knock-in (*Atp6v0a2*^*RQ/RQ*^) mice harboring the R755Q missense mutation selectively abolishing V0a2-mediated proton transport to investigate the WSS pathomechanism. Homozygous mutants from both strains displayed a reduction of growth, dermis thickness, and elastic fiber formation compatible with WSS. A hitherto unrecognized male infertility due to globozoospermia was evident in both mouse lines with impaired Golgi-derived acrosome formation and abolished mucin-type O-glycosylation in spermatids. *Atp6v0a2*^−/−^ mutants showed enhanced fucosylation and glycosaminoglycan modification, but reduced levels of glycanated decorin and sialylation in skin and/or fibroblasts, which were absent or milder in *Atp6v0a2*^*RQ/RQ*^. *Atp6v0a2*^*RQ/RQ*^ mutants displayed more abnormal migration of cortical neurons, correlating with seizures and a reduced O-mannosylation of α-dystroglycan. While anterograde transport within the secretory pathway was similarly delayed in both mutants the brefeldin A-induced retrograde fusion of Golgi membranes with the endoplasmic reticulum was less impaired in *Atp6v0a2*^*RQ/RQ*^. Measurement of the pH in the trans Golgi compartment revealed a shift from 5.80 in wildtype to 6.52 in *Atp6v0a2*^−/−^ and 6.25 in *Atp6v0a2*^*RQ/RQ*^. Our findings suggest that altered O-glycosylation is more relevant for the WSS pathomechanism than N-glycosylation and leads to a secondary dystroglycanopathy. Most phenotypic and cellular properties correlate with the different degrees of trans Golgi pH elevation in both mutants underlining the fundamental relevance of pH regulation in the secretory pathway.

**Supplementary Information:**

The online version contains supplementary material available at 10.1007/s00018-024-05506-7.

## Introduction

Many biological processes are influenced by pH levels inside cellular compartments and proton gradients across membranes are used by various secondary active transport processes [1]. The luminal pH in the secretory pathway gradually decreases from the neutral endoplasmic reticulum (ER) to the trans-Golgi compartment and secretory vesicles [2]. H^+^ concentrations are crucially dependent on the activity of vacuolar-type H^+^-ATPases (V-ATPases). V-ATPases are multisubunit complexes composed of an ATP hydrolytic part (V_1_ sector) and proton translocating membrane-embedded part (V_0_ sector) [3]. V_1_ consists of three A, B, E, G and single C, D and F subunits. V_0_ is assembled in the endoplasmic reticulum and composed of a proteolipid ring of ten c and individual a, d, e, ap1 and ap2 subunits [4].

Subunit V0a has a central role since it provides the proton channel and determines the subcellular localization of the whole V-ATPase complex [5]. It comes in four isoforms with different localizations: Subunit V0a2 was described to reside at the Golgi apparatus and early endosomes, where it seems to partially overlap with subunit V0a1, which is also found in late endosomes/lysosomes together with V0a3, while V0a4 resides within the plasma membrane [6]. As a consequence, defects in these V0a isoforms lead to different hereditary disorders: mutations in V0a1 cause epilepsy (MIM 619970) [7, 8], loss of V0a3 leads to autosomal recessive osteopetrosis (MIM 259700), V0a4 is associated with renal tubular acidosis and hearing loss (MIM 602722), and mutations in the gene *ATP6V0A2* encoding subunit V0a2 cause autosomal recessive cutis laxa type 2A (ARCL2A, MIM 219200), also called wrinkly skin syndrome (WSS).

Wrinkly and sagging skin, joint hypermobility, cortical brain malformations, epileptic seizures, growth and developmental delay, and glycosylation anomalies are hallmarks of WSS [9–11]. While the skin phenotype, due to an elastic fiber impairment, usually becomes weaker with age, the neurological phenotype can be progressive and may lead to considerable handicap [12]. Biallelic pathogenic variants in *ATP6V1E1*, encoding subunit V1E1, and *ATP6V1A*, encoding subunit V1A, cause ARCL2C (MIM 617402) and ARCL2D (MIM 617403), respectively. Although ARCL2A, -C, and -D show broad phenotypic overlaps, brain malformations and seizures seem most frequent in ARCL2A/WSS [13, 14].

The Golgi compartment is a central hub for trafficking between endoplasmic reticulum (ER), plasma membrane and the endolysosomal compartment [15]. It consists of cis, medial, and trans membranous cisternae followed by the trans-Golgi network (TGN). According to the cisternal progression model, vesicles coming from the ER form new cis cisternae while trans cisternae become dispersed into outgoing vesicles [16]. The resulting cisternal maturation of membranes is counteracted by retrograde transport vesicles ensuring that e.g. the enzymes responsible for the stepwise process of glycosylation remain in the correct position [17]. N- and O-glycosylation each have a separate set of glycosylation enzymes stepwise adding sugar residues to the glycoprotein during its travel through the ER and the Golgi compartment [18]. An early step of N-glycosylation in the Golgi is addition of fucose to the first N-acetyl glucosamine residue (core fucosylation), while terminal steps are addition of galactose and sialic acid. A special kind of O-glycosylation is the addition of glycosaminoglycans (GAGs) resulting in proteoglycans. The efficiency of glycosylation enzymes individually depends on the environment within their cisterna, e.g. the ionic composition and pH. Since individuals with V0a2 loss of function show a lack of galactose and sialic acid residues in N-glycans and reduced O-glycosylation the condition is also called ATP6V0A2-CDG (Congenital Disorder of Glycosylation) [9, 19]. The contribution of the impaired glycosylation to the disease phenotype remains incompletely understood.

In line with the main localization of subunit V0a2 at the TGN a delayed secretion of cargo proteins was shown in *ATP6V0A2*-deficient cells, but also a fragmented Golgi apparatus and a delay in brefeldin A-induced retrograde membrane trafficking from Golgi to ER [9, 11]. It was speculated that some of these observed cellular and phenotypic abnormalities might be related to functions of the V-ATPase beyond proton transport. While some experimental evidence suggested that the V0 sector could play a direct role in vesicle fusion similar to SNARE complexes, this was contradicted by other studies [20, 21]. Interactions with non-V-ATPase proteins have been described for several V-ATPase subunits. Subunit V0a2 was described to interact with endosomal ARF6 and its guanine nucleotide exchange factor ARNO in a pH-dependent fashion [22, 23].

In this study we wanted to differentiate between the function of subunit V0a2 in proton transport and additional functional aspects. To this end, we compared an *Atp6v0a2*^*−/−*^ knock-out with an *Atp6v0a2*^*RQ/RQ*^ knock-in model harboring the R755Q mutation leading to a V0a2-RQ protein incapable of proton translocation. Both models broadly recapitulate the WSS phenotype, but show differences in organ involvement and glycosylation deficiencies, correlating to different degrees of Golgi pH and vesicular trafficking disturbance. We also depict a hitherto unrecognized defect in acrosome formation leading to male infertility.

## Results

### Growth retardation and a thinned dermis with reduced elastic fibers as hallmarks of WSS in ***Atp6v0a2***^***−/−***^ and ***Atp6v0a2***^***RQ/RQ***^ mouse mutants

**Fig. 1:**
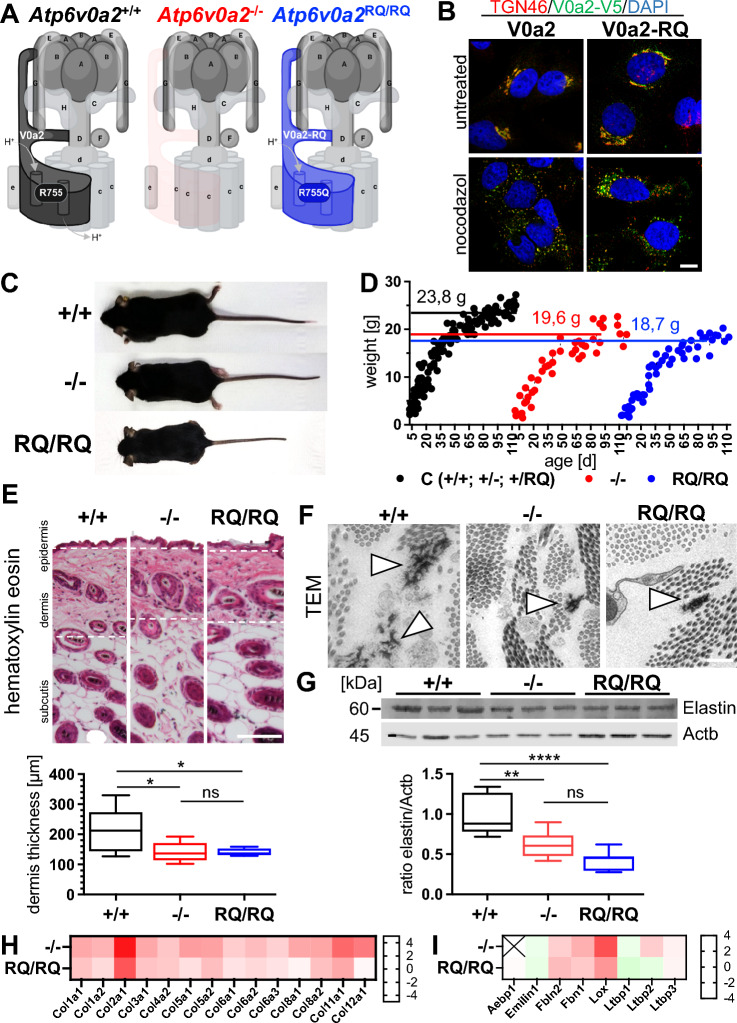
Growth retardation, thinned skin with reduced elastic fibers as hallmarks of WSS, and reduced ECM protein production by fibroblasts in *Atp6v0a2*^*−/−*^ and *Atp6v0a2*^*RQ/RQ*^ mouse mutants. **A** Illustration of the consequences of *Atp6v0a2*^*−/−*^ knock-out (red) and *Atp6v0a2*^*RQ/RQ−*^ knock-in (blue) on the V-ATPase multiprotein complex. Amino acid arginine 755 exchanged for glutamine (R755Q) in knock-in *Atp6v0a2*^*RQ/RQ*^ is located in the proton channel of the V0a2 subunit. Adapted from "Structure of V-ATP Synthase", by BioRender.com (2023). Retrieved from https://app.biorender.com/biorender-templates. **B** Mild retroviral overexpression of V0a2-V5 and V0a2-RQ-V5 (green) in HeLa cells. Immunofluorescence staining revealed co-localization of wildtype and R755Q harboring subunit V0a2-RQ with trans-Golgi network marker TGN46 (red). Nuclei were stained with DAPI (blue). Separation of the Golgi stacks was induced by nocodazol. Scale: 10µm. **C** *Atp6v0a2*^*−/−*^ and *Atp6v0a2*^*RQ/RQ*^ animals with wildtype littermates at five weeks of age. **D** Body weight of female *Atp6v0a2*^*−/−*^ (−/−; red) and *Atp6v0a2*^*RQ/RQ*^ (RQ/RQ; blue) mice compared with their wildtype (+ / +) and heterozygous (± ; + /RQ) littermates (black). Numbers denote the mean weight at age 100–110 days (n = 5 mutants and littermate controls). **E** Hematoxylin–eosin staining of skin sections from five weeks old animals demonstrating a reduction of dermis thickness (indicated by dotted lines). Dermis thickness was measured using Inkscape. Scale: 100 µm; n = 6–8 animals per genotype. **F** Ultrastructural analysis of dermis from five weeks old animals showing reduced elastic fibers (white arrowheads). Scale: 500 nm. **G** Immunoblot of elastin (Eln) relative to actin beta (Actb) lysates of skin from five weeks old mice, n = 3 animals per genotype. Statistical analysis by one-way ANOVA. **H** Log2 changes of collagen proteins and **I** proteins relevant for elastic fiber formation in mouse embryonic fibroblasts (MEFs) analyzed by mass spectrometry. Aebp1 was not detected in *Atp6v0a2*^*−/−*^. P * < 0.05; ** < 0.01; **** < 0.0001; ns: not significant

In order to dissect the molecular pathomechanism of WSS/ARCL2A we generated two different mouse models: 1. A knock-out by deletion of exons 15 to 17 of *Atp6v0a2* and 2. A knock-in harboring the mutation c.2263_2624delinsCA in exon 18 leading to the amino acid substitution R755Q (Fig. 1A) (Figs. S1 and S2). This substitution interrupts the interaction of V0a2 with the V0c subunits, thus disturbing proton transport of the v-ATPase complex [20]. Since a correct subcellular distribution of the mutant V0a2-RQ is prerequisite for an integration into the v-ATPase complex, we verified the physiological Golgi localization by retroviral expression in Hela cells (Fig. 1B).

*Atp6v0a2*^*−/−*^ mice showed a reduction of overall *Atp6v0a2* mRNA levels by 80%, whereas the *Atp6v0a2*^*RQ/RQ*^ mRNA was stably expressed (Fig. S3A and B). Immunoblotting of brain homogenates showed a complete loss of V0a2 in *Atp6v0a2*^*−/−*^ and slightly reduced protein levels in *Atp6v0a2*^*RQ/RQ*^ (Fig. S3C and D). Due to variable functionality of V-ATPase antibodies in tissue lysates we investigated the levels of other relevant V-ATPase subunits in membrane preparations from mouse embryonic fibroblasts (MEFs). Again, V0a2 was absent in *Atp6v0a2*^*−/−*^ and somewhat reduced in *Atp6v0a2*^*RQ/RQ*^ (Fig. S3E). The only other significant alteration compared to wildtype was a roughly 50% enhanced V0a1 level in *Atp6v0a2*^*RQ/RQ*^, whose broad subcellular localization overlaps with V0a2.

Homozygous *Atp6v0a2*^*−/−*^ and *Atp6v0a2*^*RQ/RQ*^ mice were slightly smaller than wildtype and heterozygous littermates (Fig. 1C). Both mutants showed a reduced body weight (Fig. 1D). Given that a dominant negative effect of the paralogous mutation R740S in subunit V0a3 was suggested it is important to mention that *Atp6v0a2*^+*/RQ*^ mutants were indistinguishable from wildtype animals [24]. While the skin’s outward appearance was not altered, a reduced dermis thickness in *Atp6v0a*2^−/−^ and *Atp6v0a2*^*RQ/RQ*^ mice recapitulated the skin phenotype of WSS (Fig. 1E). Ultrastructural analysis of the thinned dermis revealed smaller elastic fibers (Fig. 1F). By immunoblotting of whole skin lysates, we detected similarly reduced elastin (Eln) levels in both mutants (Fig. 1G). Thus, mildly reduced longitudinal growth and thinned skin with reduced elastin content as hallmarks of WSS were reproduced in both mouse models.

We wanted to know whether the observed skin phenotype was due to altered secretion of extracellular matrix (ECM) proteins. To this end, the proteome of cultured mouse embryonic fibroblasts (MEFs) was analyzed by label-free proteomics (Tables S1 and S2). We found reduced levels of several collagens (Fig. 1H), but only milder changes in proteins relevant for elastic fiber formation (Fig. 1I). For a broader comparison we performed gene set enrichment analysis (GSEA), which revealed significant negative enrichment of protein groups related to ECM and collagen formation for *Atp6v0a2*^*−/−*^, while no significant enrichment was observed for *Atp6v0a2*^*RQ/RQ*^ (Table S3). This implied a perturbation of ECM production upon loss of V0a2, which was milder in *Atp6v0a2*^*RQ/RQ*^.

### Increased fucosylation of glycoproteins and decorin overmodification, but reduced sialylation in skin and fibroblasts hint to differential effects of V0a2 loss of function.

**Fig. 2:**
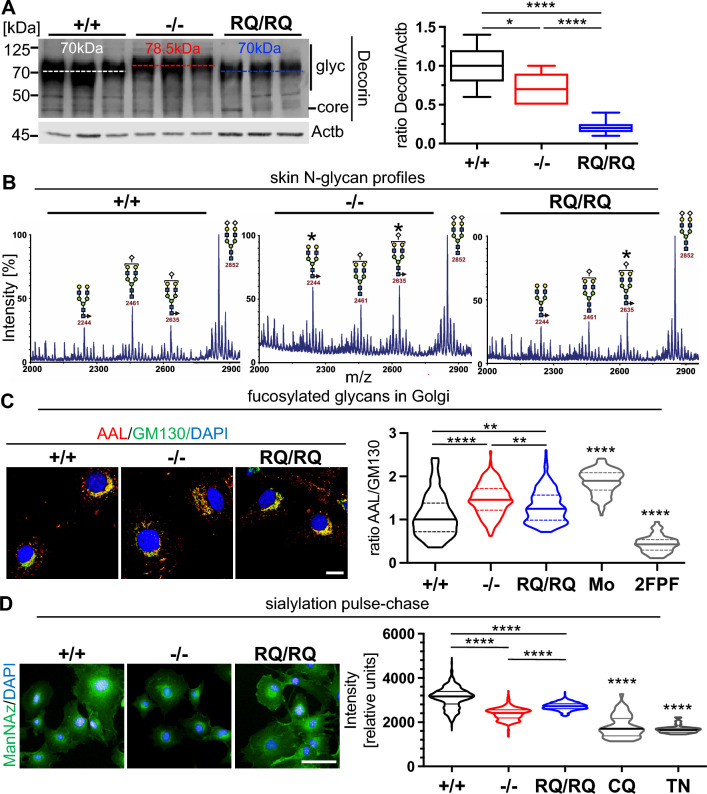
Increased fucosylation of glycoproteins and decorin overmodification, but reduced sialylation in skin and fibroblasts hint to differential effects of V0a2 loss of function. **A** Immunoblot of decorin (Dcn) in protein lysates of skin from five week old mice relative to actin beta (Actb), n = 3 animals per genotype. Increased modification of Dcn in the *Atp6v0a2*^*−/−*^ mouse model is indicated by upward shift of the glycanated band. Statistical analysis by one-way ANOVA. **B** N-glycan analysis of mouse skin by mass spectrometry (average of n = 3 per genotype). Peaks at 2244 and 2635 m/z (*) representing core fucosylated species are elevated in *Atp6v0a2*^*−/−*^; *Atp6v0a2*^*RQ/RQ*^ shows only an altered 2635 peak. Sugar residue symbols: blue squares: N-acetyl glucosamine, green circles: mannose, red triangles: fucose, yellow circles: galactose, white checks: sialic acid. **C** Staining of MEFs with Aleuria Aurantia lectin (AAL, red) detecting fucosylated glycans. Representative images of localization of AAL signals in the Golgi compartment as labeled by GM130 (green) in *Atp6v0a2*^*−/−*^ (−/−), and *Atp6v0a2*^*RQ/RQ*^ (RQ/RQ) MEFs. Scale: 10µm. The control cells (+ / +) were treated with the anterograde transport blocker monensin (Mo) or with the fucosyltransferase inhibitor 2F-peracetyl-fucose (2FPF). The ratio of fluorescence intensity of AAL/GM130 of at least 100 cells per cell line (n = 3 per genotype) was quantified. Statistical analysis by Kruskal–Wallis test. **D** Analysis of sialylation in vitro. MEFs were labeled for 6 h with N-Azidoacetyl-mannosamine (ManNAz)(green). Nuclei were stained with DAPI (blue). Quantification of the fluorescence signal of 60–100 cells from n = 3 cell lines per genotype. Median fluorescence intensities of ManNAz in the Golgi compartments show a reduced transfer of sialic acid within the Golgi apparatus of *Atp6v0a2*^−/−^and *Atp6v0a2*^*RQ/RQ*^ MEFs. Treatment with tunicamycin (TN) and chloroquine (CQ) were used as negative controls. Statistical analysis by Kruskal–Wallis test. Scale: 50 µm; P * < 0.05; ** < 0.01; **** < 0.0001; ns: not significant

We wanted to learn more about a possible role of glycosylation changes in the observed skin phenotype. Glycanated decorin (Dcn), a commonly analyzed indicator proteoglycan, was less abundant in skin lysates from both mutants. Surprisingly, decorin showed a higher molecular weight (78.5 kDa) in *Atp6v0a2*^*−/−*^ mice compared to *Atp6v0a2*^*RQ/RQ*^ and wildtype mice (70 kDa), indicating elongated or excessively sulfated GAG chains (Fig. 2A). A similar upward shift of the glycanated band was detected for decorin secreted by MEFs in vitro (Fig. S4). Next, we analyzed skin N-glycans by mass spectrometry, which showed no overall reduction in both mutant lines. However, both detected biantennary fucosylated N-glycan species were more abundant in *Atp6v0a*2^−/−^ skin, while in *Atp6v0a2*^*RQ/RQ*^ only one biantennary N-glycan species was mildly elevated (Fig. 2B). A similar increase of a fucosylated species was visible in serum N-glycans (Fig. S5).

We hypothesized that the increased core fucosylation could be either a sign for a longer retention of glycoproteins or increased activity of the responsible glycosyltransferase Fut8 in the Golgi apparatus. To test this, we stained MEFs with the Aleuria Aurantia lectin (AAL) that recognizes fucose residues and compared the fluorescence intensity to the Golgi marker GM130 (Fig. 2C). Relative AAL intensity in the Golgi area was highest in *Atp6v0a2*^*−/−*^ MEFs, followed by *Atp6v0a2*^*RQ/RQ*^ and control cells. Control cells incubated with the anterograde transport blocker and proton ionophore monensin (Mo) as positive control showed the highest relative AAL intensity and the fucosyltransferase inhibitor 2FPeracetyl-Fucose (2FPF) entailed a steep drop of intensity. Additional assays showed a non-significant trend towards stronger fucosylation in whole cell lysates, but lower fucosylation of surface glycoproteins in *Atp6v0a2*^*−/−*^ (Fig. S6)*.*

*ATP6V0A2* deficiency leads to reduced sialylation of N-glycans [9]. Although N-glycan profiles from skin (Fig. 2B) or serum (Fig. S5) did not show corresponding changes, we speculated that a reduced sialylation capacity might be uncovered in a pulse-chase experiment. To this end, we pulsed MEFs with tetraacetylated N-Azidoacetyl-mannosamine (ManNAz), which is converted into sialic acid and can be labeled through click-chemistry. As positive controls chloroquine (CQ) and the N-glycosylation blocker tunicamycin (TN) were used. Here, a mirror image of the abovementioned AAL labeling is seen: *Atp6v0a2*^*−/−*^ MEFs displayed the strongest reduction and *Atp6v0a2*^*RQ/RQ*^ cells were intermediate (Fig. 2D).

In summary, *Atp6v0a2* deficiency entails reduced levels of glycanated decorin and sialylated glycoproteins. Paradoxically, decorin glycosaminoglycan modification and N-glycan fucosylation are increased primarily in *Atp6v0a2*^*−/−*^. Except for decorin glycanation the mentioned effects were milder or absent in *Atp6v0a2*^*RQ/RQ*^.

### Differential effects of V0a2 deficiency on Golgi trafficking and pH regulation

**Fig. 3:**
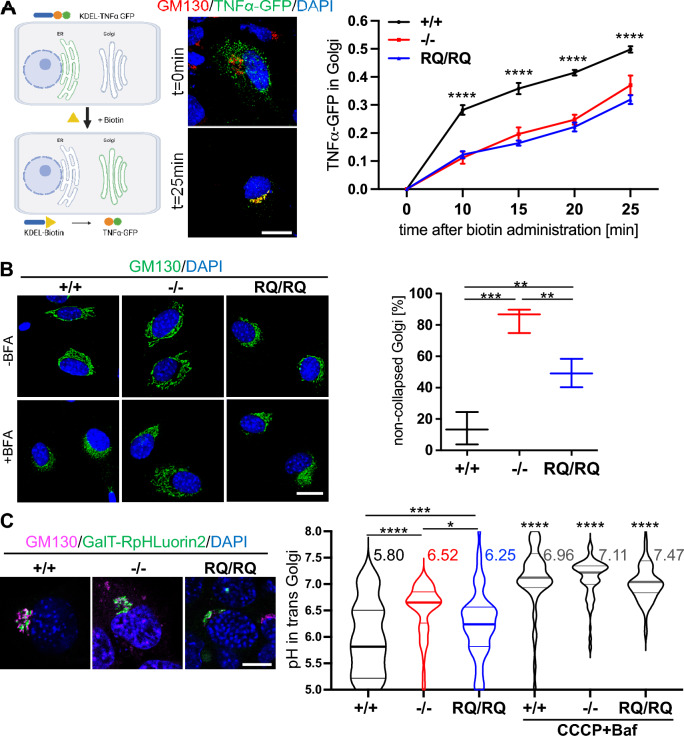
Differential effects of *Atp6v0a2* knock-out and knock-in on transport within the secretory pathway and pH in the trans Golgi compartment. **A** Analysis of intracellular trafficking using the Str-KDEL-IRES-TNFa-GFP-RUSH construct, which was transiently transfected into immortalized MEFs. Representative images of wildtype MEFs show Golgi marker GM130 (red) and TNFα-GFP (green) before and after 25 min biotin treatment. Analysis by two-way ANOVA. Scale: 20µm. Graphical illustration created with BioRender.com. **B** Analysis of Golgi collapse after brefeldin A (BFA) treatment. Golgi morphology was analyzed using the Golgi marker GM130 (green). Quantification of non-collapsed Golgi apparatus in n = 3 cell lines per genotype. Golgi collapse is less delayed in *Atp6v0a2*^*RQ/RQ*^ MEFs when compared to *Atp6v0a2*^*−/−*^. Statistical analysis by one-way ANOVA. Scale: 20 µm. **C** Measurement of pH in the trans Golgi compartment using GalT-RpHLuorin2 transfected into immortalized MEFs (n = 3 independent cell lines per genotype). Left images show the localization of the GalT-RpHLuorin2 probe in trans Golgi compartment in all three genotypes. In the violin plot of the measurement results the bold lines represent the median, means are given at the top of the violin representation. An increased Golgi pH in *Atp6v0a2*^*−/−*^ (mean 6.52) and *Atp6v0a2*^*RQ/RQ*^ (mean 6.25) compared to controls (mean 5.80) was visible. MEFs treated with the proton ionophore carbonyl cyanide m-chlorophenyl hydrazone (CCCP) and bafilomycin A1 (Baf) served as positive controls. Scale = 10 µm. Statistical analysis by one-way ANOVA. P * < 0.05; ** < 0.01; *** < 0.001; **** < 0.0001

We wondered whether the increased core fucosylation and decorin glycosaminoglycan modification might be correlated to an intracellular retention. Therefore, we applied the retention using selective hooks (RUSH) system to investigate the intracellular protein trafficking of the glycoprotein tumor necrosis factor (TNFα) [25]. After biotin treatment of the cells TNFα-GFP was released from endoplasmic reticulum (ER)-localized KDEL, which induced its anterograde transport to the Golgi. This analysis revealed a strong delay in protein cargo trafficking between the ER and the Golgi and an increased intracellular retention of proteins in *Atp6v0a2*^*−/−*^ and *Atp6v0a2*^*RQ/RQ*^ MEFs (Fig. 3A) (Fig. S7).

By blocking ADP ribosylation factor 1 (ARF1) function leading to a loss of the COPI coat Brefeldin A (BFA) perturbs Golgi transport and induces an abnormal retrograde transport leading to fusion of Golgi cisternae with the ER [26]. This was recapitulated in MEFs by an increased co-localization of the Golgi marker GM130 with the ER marker calreticulin after BFA addition mirrored in a ten-fold increased Pearson correlation coefficient (Fig. S8). *ATP6V0A2*-deficient fibroblasts showed a delay of this BFA-induced effect [9]. After 8 min BFA incubation *Atp6v0a2*^*−/−*^ MEFs displayed a delayed response with 87% intact Golgi cisternae versus 14% in control MEFs (Fig. 3B). In contrast, this delay was significantly weaker in *Atp6v0a2*^*RQ/RQ*^ MEFs with only 50% non-collapsed Golgi compartments. We also performed a BFA washout assay. *Atp6v0a2*^*−/−*^ and *Atp6v0a*^*RQ/RQ*^ MEFs showed reduced Golgi re-assembly with 10% assembled Golgi compartments compared to 40% in wildtype after 30 min (Fig. S9). This result underlines the abovementioned RUSH assay results.

Finally, we wanted to know in how far the observed abnormalities of glycosylation and trafficking might correlate with pH dysregulation in the Golgi apparatus. To this end, immortalized MEFs were transiently transfected with GalT-RpHLuorin2, which directs the pH-sensitive probe to the trans Golgi, and with GPI-anchored RpHLuorin2 targeting the cell surface as a calibrator [27]. Fluorescence intensities of both probes after excitation at 405 and 488 nm were compared and pH deduced through a calibration curve. In wildtype cells we detected a mean pH of 5.80 ± 0.89 SD, which is within the described range. In contrast, *Atp6v0a2*^*−/−*^ cells showed a clearly elevated pH (mean 6.52 ± 0.47 SD), which was less pronounced in *Atp6v0a2*^*RQ/RQ*^ cells (mean 6.25 ± 0.88 SD) (Fig. 3C). Bafilomycin (Baf) and carbonyl cyanide m-chlorophenyl hydrazone (CCCP) treatment raised the mean pH to high levels between 6.96 and 7.47.

The observed more pronounced pH changes in *Atp6v0a2*^*−/−*^ and *Atp6v0a2*^*RQ/RQ*^ correlate to the decorin band shift, the enhanced fucosylation, the reduced sialylation, and the delayed BFA effects. In contrast, the more strongly reduced overall abundance of glycanated decorin in *Atp6v0a2*^*RQ/RQ*^ does not correlate and might therefore be due to an additional dysfunction induced by the R755Q mutation in V0a2.

### Overmigration of cortical neurons as a result of impaired α-dystroglycan O-mannosylation

**Fig. 4:**
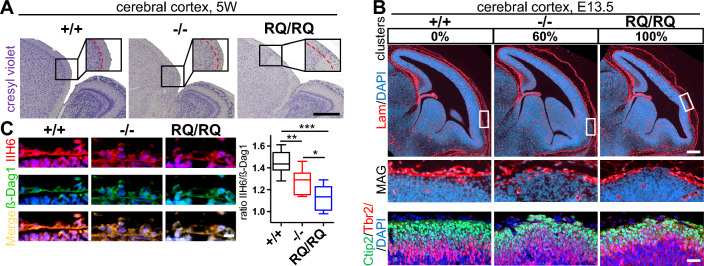
An O-glycosylation defect of α-dystroglycan in the neocortex leads to overmigration of neurons. **A** Overmigration of neurons in the cerebral cortex of *Atp6v0a2*^*−/−*^ and *Atp6v0a2*^*RQ/RQ*^ mice at the age of five weeks. Red line mark clusters of overmigrated neurons. Scale: 500 µm. **B** Top: Numbers give the percentages of embryos in which ectopic neuron clusters were found in serial sections in neocortex of E13.5 embryos. Local gaps in the basal lamina are visible (white box) in sections stained with laminin (Lam; red) and DAPI (blue). Scale: 200 µm. Bottom: Immunofluorescence staining for Ctip2 (green) for lower layer cortical neurons and Tbr2 (red) for subventricular intermediate neuronal progenitors. Nuclei were stained with DAPI (blue). The overmigrated neurons express the marker Ctip2 and thus originate from the cortical plate. Subventricular neurons are found in the upper layer. Scale: 100 µm. **C** Reduction of α-dystroglycan (α-Dag1) O-mannosylation at the pial basement membrane in E13.5 embryos. Left: immunostaining of glyco-epitope IIH6 (red), β-Dag1 (green) of piabasal membrane. Scale: 10 µm. Right: relative fluorescence intensity of IIH6 in relation to β-Dag1 for n = 5 animals per genotype. Statistical analysis by one-way ANOVA. P * < 0.05; ** < 0.01; *** < 0.001

Common features of WSS are developmental delay and seizures [12]. In brain imaging a frontoparietal cobblestone lissencephaly of varying severity can be observed in nearly all patients [28]. Lissencephaly is the result of an abnormal neuronal migration during cerebral cortex development. Investigating the cortical layering in postnatal developmental stages in both mouse models we found a neuronal overmigration of neurons into layer I of the cerebral cortex, which was more pronounced in *Atp6v0a2*^*RQ/RQ*^ mice (Fig. 4A). Like in the human disorder the predilection site of these changes was the frontal cortex. These findings correlated with an incidence for seizures of 21% (n = 6/28) of *Atp6v0a2*^*−/−*^ and 50% (n = 8/16) of *Atp6v0a2*^*RQ/RQ*^ animals. Ectopic neuronal clusters in the cerebral cortex were already evident at prenatal stage E13.5 in 3 of 5 *Atp6v0a2*^*−/−*^ and 5 of 5 *Atp6v0a2*^*RQ/RQ*^ embryos (Fig. 4B) (Fig. S10), again indicating a stronger phenotype of the *Atp6v0a2*^*RQ/RQ*^ mouse model. Immunofluorescence staining showed abnormal layering of Tbr2^+^ intermediate progenitor and Ctip2^+^ lower layer cortical neurons (Fig. 4B).

Precursors of cortical neurons migrate radially along a glial scaffold and are finally stopped by the pial basement membrane [29]. In both mouse mutants the basal lamina, a part of the pial basement membrane, appeared disrupted in areas of ectopic neuronal clusters (Fig. 4B). The contact between neurons and basement membrane is mediated by α-dystroglycan (α-Dag1), the central component of the dystrophin–glycoprotein complex binding to laminin through O-mannosyl glycans recognized by the IIH6 antibody [30]. A reduction of the IIH6 immunofluorescence intensity in relation to ß-dystroglycan as a glycosylation-independent reference was observed in *Atp6v0a2*^*−/−*^* and Atp6v0a2*^*RQ/RQ*^ mice indicating an O-mannosylation deficiency (Fig. 4C). We excluded a Gpr56/ type III collagen-dependent mechanism for cobblestone-like cortical malformations through immunoblotting (Fig. S11). Interestingly, glycan profiling excluded a role of N-glycosylation alterations in the brain phenotype (Fig. S12). In conclusion, we identified a secondary dystroglycanopathy in *Atp6v0a2*^*−/−*^ and *Atp6v0a2*^*RQ/RQ*^ mice in absence of a N-glycosylation deficiency.

### Male infertility and globozoospermia in ***Atp6v0a2***^−/−^ and ***Atp6v0a2***^***RQ/RQ***^ mice

**Fig. 5:**
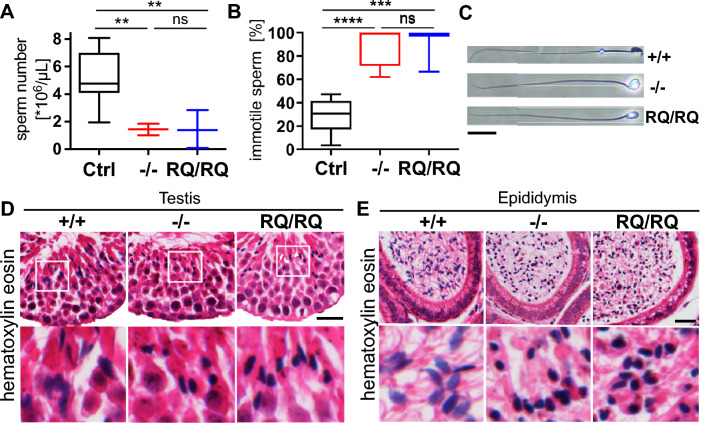
*Atp6v0a2* deficient male mice display globozoospermia. Determination of sperm numbers (**A**) and motility (**B**) of spermatozoa from the epididymis of three months old mice n (Ctrl= + / + , +/− , + /RQ) = 11, n (−/−) = 4, n (RQ/RQ) = 3. Sperm counts and motility were dramatically reduced in *Atp6v0a2*^*−/−*^ and *Atp6v0a2*^*RQ/RQ*^ mice. **C** Phase contrast image of spermatozoa from the cauda epididymidis of adult mice. Note rounded heads of spermatozoa from *Atp6v0a2*^*−/−*^ and *Atp6v0a2*^*RQ/RQ*^ animals. Scale = 20 µm. **D** Testis and **E** epididymis sections were stained with hematoxylin and eosin showing abnormal round shape of sperm heads in both mutants. Scale: 20 µm. Statistical analysis by one-way ANOVA. P ** < 0.01; *** < 0.001; **** < 0.0001; ns: not significant

While homozygous *Atp6v0a2*^−/−^ and *Atp6v0a2*^*RQ/RQ*^ females reproduced normally, their male counterparts failed to produce any offspring. In contrast, matings of heterozygous males and females from both mouse lines resulted in normal litter frequencies and sizes (6.9 ± 1.7 for knockout and 6.8 ± 1.4 for knock-in). The testes of homozygous *Atp6v0a2* mice had normal size and morphology (Fig. S13). Spermiogram analysis of epididymal spermatozoa showed an 80% reduction of total sperm numbers (Fig. 5A) and 80–90% immotile sperm compared to 20–30% in control mice (Fig. 5B). Sperm motility can be impaired by malformations of the heads or the flagellum [31]. In light microscopy epididymal spermatozoa from both mutants appeared to have normal flagella but round-shaped heads (globozoospermia) compared to the typical hook-shaped wildtype spermatozoa (Fig. 5C). This abnormal morphology was evident already during spermatogenesis in testis (Fig. 5D) and also in the lumen of the epididymis in the mutants (Fig. 5E). Otherwise, the testicular and epididymal architectures were normal.

### Acrosomal maturation defects in ***Atp6v0a2***^−/−^ and ***Atp6v0a2***^***RQ/RQ***^ mice and a possible contribution of Gopc

**Fig. 6:**
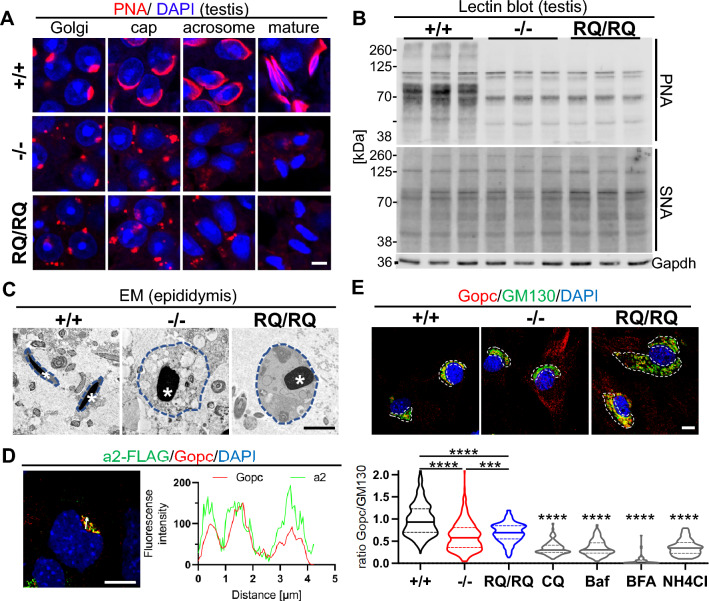
Acrosome formation and O-GalNac synthesis defect in *Atp6v0a2* mutants and reduced Gopc Golgi recruitment. **A** Acrosomes were stained by PNA lectin (red) and nuclei by DAPI (blue) in testis sections from three months old mice. Representative images are shown for the four phases (Golgi, cap, acrosome, and mature) of acrosome maturation. *Atp6v0a2*^*−/−*^ and *Atp6v0a2*^*RQ/RQ*^ testis show acrosomal vesicles during the Golgi phase, which do not fuse. Fragmented acrosomes were detected in the acrosome phase but not in the maturation phase. Scale = 20 µm. **B** Reduced O-glycosylation in testis. Blots of testicular lysates from three months old mice (n = 3 per genotype) stained with lectins PNA and SNA. **C** Transmission EM images showing spermatozoa in the epididymis. Note accumulation of translucent vesicles in *Atp6v0a2*^*−/−*^ mutants that are absent in controls and *Atp6v0a2*^*RQ/RQ*^. The round shaped sperm head is comparable in both mutants. Scale = 2,5 µm. **D** Co-localization of Gopc and transiently expressed H^+^-ATPase subunit a2 with flag-tag in immortalized *Atp6v0a2*^+*/*+^ MEFs. The strong overlap of both proteins in the Golgi region (white arrow) is demonstrated by the overlapping fluorescense intensity curves. Scale: 10 µm. **E** Reduced recruitment of Gopc to the Golgi apparatus in *Atp6v0a2*^*−/−*^ and *Atp6v0a2*^*RQ/RQ*^ MEFs. Representative images show the localization of Gopc (red) in the Golgi compartment (dotted white line) as labeled by GM130 (green). Treatment with chloroquine (CQ), bafilomycin (Baf), brefeldin A (BFA), and ammonium chloride (NH4Cl) were used as controls. Scale bar: 10 µm. Bottom: The ratios of overlapping fluorescence intensity of Gopc/Gm130 from at least 100 cells per cell line (n = 3 per genotype) were quantified. Statistical analysis by Kruskal–Wallis test. P *** < 0.001; **** < 0.0001

A central process in spermiogenesis is the formation of the acrosome, which contains proteases necessary for the penetration of the zona pellucida [32]. It is mainly formed by transport of Golgi-derived vesicles to the perinuclear region, where they fuse and form a large membranous sac covering the anterior part of the nucleus. Subunit V0a2 expression in the acrosome of human motile sperm has been described [33]. Due to its high content of glycoproteins the acrosome can be stained by peanut agglutinin (PNA), which recognizes mucin-type O-glycans with Gal-β(1–3)-GalNAc bond. Beginning in the Golgi phase of acrosome formation the Golgi cisternae appeared fragmented in *Atp6v0a2*^−/−^ and *Atp6v0a2*^*RQ/RQ*^ mutants (Fig. 6A) (Fig. S14). Thus, an acrosomal cap did not form and in the maturation phase only a diffuse PNA-staining was evident. Given these diffuse acrosomal PNA lectin signals in *Atp6v0a2* mutants we also investigated mature spermatozoa in the epididymis that showed 60–70% weaker PNA signals (Fig. S15). We next investigated whether this glycosylation defect was generalized using lectin blots of whole testis lysates (Fig. 6B). Whereas the reduced PNA labeling intensity in *Atp6v0a2*^−/−^ and *Atp6v0a2*^*RQ/RQ*^ confirmed the histological PNA-stainings, Sambucus nigra (SNA) lectin, which recognizes terminal sialic acids on N- and O-glycans, gave comparable signals.

Ultrastructural analyses of spermatozoa revealed further insights into the exact nature of the impaired acrosome formation (Fig. 6C) (Fig. S16). Round shaped nuclei, excess cytoplasm, and abnormal insertion and morphology of the sperm tails were evident, which are all typical signs for globozoospermia. In both mutants acrosomal vesicles lacked electron dense material. While high numbers of proacrosomal vesicles were formed in *Atp6v0a2*^−/−^ these vesicles were fewer and smaller in *Atp6v0a2*^*RQ/RQ*^.

Defects in Gopc, a trans-Golgi protein influencing in- and outgoing vesicle trafficking, cause globozoospermia [34]. In testis lysates the levels of Gopc and the interacting protein Pick1 were not reduced in mutants compared to controls (Fig. S17). Immunofluorescence staining of overexpressed V0a2 and Gopc/GOPC in MEFs and human skin fibroblasts (HAF) indicated a strong co-localization in both species (Fig. 6D) (Fig. S18). Also in control MEFs Gopc was readily detected at the Golgi cisternae (Fig. 6E). However, the Gopc immunofluorescence signals at the Golgi apparatus were less intense in *Atp6v0a2*^−/−^ and *Atp6v0a2*^*RQ/RQ*^ MEFs as well as in control cells incubated with alkalizing agents (Fig. 6E). Importantly, the overall Gopc protein levels were not affected by the elevated Golgi pH (Fig. S19). This points to a reduced pH-dependent Gopc recruitment to Golgi membranes in *Atp6v0a2*-deficient cells, which may contribute to the observed block of acrosome formation.

## Discussion

The *Atp6v0a2*^−/−^ and *Atp6v0a2*^*RQ/RQ*^ mouse models both recapitulate the ARCL2A/WSS/ATP6V0A2-CDG phenotype with cutis laxa-like skin changes due to reduced elastin deposition. Abnormal migration of cortical neurons correlated with impaired α-dystroglycan O-mannosylation. In addition, we describe a defect of acrosome formation leading to globozoospermia as a novel phenotype.

All forms of cutis laxa and related connective tissue disorders go along with abnormal dermal elastic fibers [35]. In both analyzed *Atp6v0a2* mouse models elastic fibers were rarefied and elastin levels were reduced in the skin. Elastic fibers consist of a microfibrillar scaffold onto which amorphous elastin is loaded [36]. While tropoelastin was not reliably detected in the mutant MEF proteomes several elastin-associated proteins were less abundant. A Golgi accumulation of tropoelastin induced by bafilomycin A1 and in fibroblasts from individuals with *ATP6V0A2* defects was described suggesting that pH dysregulation impairs physiological tropoelastin deposition [11, 37]. We also observed a reduced abundance of glycanated decorin. Since proteoglycans like decorin and glycosaminoglycans (GAG) can influence formation of the microfibrillar network and human connective tissue disorders have been linked to defects in GAG synthesis the decorin changes possibly also contribute to the elastic fiber reduction [38–40].

The loss of subunit V0a2 did not globally impair glycosylation processes, but even elevated fucosylation of N-glycans and length or sulfation of GAG attached to the decorin core protein. Core fucosylation mediated by the α1,6-fucosyltransferase FUT8 is a relatively early step of N-glycosylation before the addition of galactose and sialic acid [41]. In fact, sialylation even inhibits core fucosylation [42]. Therefore, FUT8 is active in the less acidic cis and medial cisternae, not in the trans cisternae where V0a2 resides, whose loss primarily affects the addition of terminal galactose and sialic acid in humans [9]. A delay of anterograde trafficking in the secretory pathway as shown by the RUSH assay might lead to enhanced core fucosylation through a longer exposure to FUT8. A similar mechanism is probably behind the increased GAG modification of decorin. The less abnormal pH and retrograde transport in the Golgi in *Atp6v0a2*^*RQ/RQ*^ might suppress these effects and explain the milder impairment of sialylation in vitro compared to *Atp6v0a2*^−/−^.

During brain development the migration of cortical neurons is directed by a glial cell scaffold and the pial basement membrane [29]. O-mannosylation of α-dystroglycan is important for mediating the contact between migrating neurons and laminins within the basement membrane. This is illustrated by cortical layering defects in individuals with mutations in the glycosylation enzyme O-mannosyl-transferase (POMT) or the xylosyl- and glucuronyltransferase 1 LARGE [43]. However, these brain malformations are global and more dramatic than those observed in WSS. It is currently unclear why the overmigration in *Atp6v0a2* mutant animals occurs in a focal fashion and mostly in the frontal cortex. Possibly, neurons lacking V0a2 need an additional trigger perturbing α-dystroglycan O-mannosylation to a degree that locally prevents ordered migration. A whole-embryo single-cell sequencing analysis of E13.5 embryos revealed reduced numbers of neuronal progenitors and floor plate cells in both *Atp6v0a2* mutants [44]. How this alteration is related to V-ATPase function at the Golgi function and in how far it may contribute to the observed structural brain anomaly requires further investigations.

The previously described expression of the V0a2 subunit in principal and clear cells of the murine epididymis and in human spermatozoa suggested a role in fertility [33, 45]. Both *Atp6v0a2* mutants show infertility due to globozoospermia, which affects 0.1% of infertile men. Several murine and human genetic defects causing globozoospermia affect Golgi proteins involved in acrosome formation, but variants in *ATP6V0A2* have not yet been described to affect this process [32, 46, 47]. This may have several reasons: (i) Most of the few described individuals affected by WSS were investigated before entering the reproductive age and (ii) due to their syndromic appearance these individuals would probably be excluded from genetic studies on isolated male infertility. Therefore, abnormal spermatogenesis in male individuals with biallelic pathogenic variants in *ATP6V0A2* may have been overlooked.

Besides *Atp6v0a2*, the only other known gene causing globozoospermia associated with ion homeostasis is *Slc9a8*, encoding Na^+^/H^+^-exchanger 8 [48]. The importance of the proper pH regulation in proacrosomal vesicles is furthermore underlined by the spermiogenesis disruption after injection of alkalizing agents [49, 50]. Defects in the interacting proteins Gopc and Pick1 impair the transport of Golgi vesicles to the acrosome and their recycling, leading to globozoospermia [34, 51]. Interestingly, the recruitment of Gopc to Golgi membranes was impaired in both *Atp6v0a2* models, but the overall levels of this protein were not reduced. Gopc modulates several aspects of vesicle trafficking at the trans-Golgi [52–54]. However, the degree of the Gopc loss from the Golgi (about 50%) alone is not sufficient to explain the acrosome abnormality. In testis, mucin-type O-glycosylation is most abundant in spermatids and spermatozoa. The only N-acetylgalactosamine transferase expressed by spermatids and spermatozoa is GALNT3, which localizes to the Golgi compartment. Strikingly, *Galnt3* knockout mice display globozoospermia and a failure of acrosome formation similar to our *Atp6v0a2* mutants [55]. We therefore propose that the aberrant Golgi homeostasis induced by the loss of function of V0a2 leads to an O-glycosylation defect through an impairment of GALNT3. The link between mucin-type O-glycosylation and acrosome formation remains to be identified [55, 56].

Our mouse mutants are the first murine models for V-ATPase-related cutis laxa syndromes, and complement previous findings in two zebrafish mutants deficient for subunit V1E1 showing Golgi as well as lysosomal alterations [57]. Results are difficult to compare since loss of V1E1 affects V-ATPase complexes in all cellular compartments and the mutants were lethal at the larval stage.

**Fig. 7:**
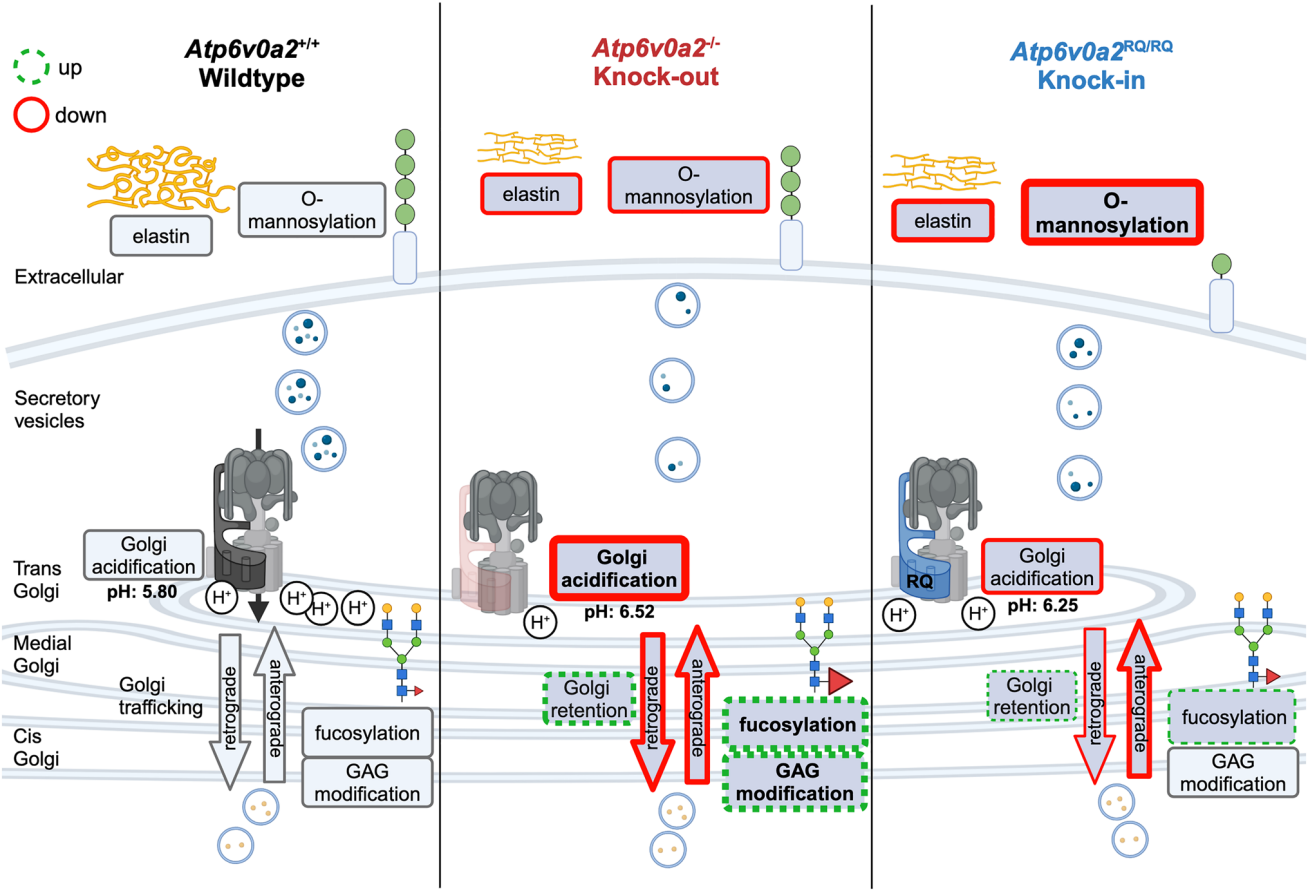
Schematic summary of pathomechanism in *Atp6v0a2* knock-out and proton channel-impaired R755Q knock-in mouse models. The V0a2 subunit encoded by *Atp6v0a2* mostly resides in the trans Golgi apparatus. To dissect the contributions of proton transport and Golgi-dependent glycosylation defects to the pathomechanism of wrinkly skin syndrome (WSS) we compared a murine knock-out (*Atp6v0a2*^−/−^) of subunit V0a2 with a knock-in of mutation R755Q (*Atp6v0a2*^*RQ/RQ*^) selectively blocking proton transport. Thinned skin with smaller elastic fibers as well as acrosome formation defects leading to globozoospermia were overall similar in both mutants. While the knock-out led to stronger abnormalities in N-glycan fucosylation and glycosaminoglycan (GAG) modification the knock-in showed a more strongly impaired O-glycosylation in the brain leading to abnormal neuronal migration and frequent seizures. These findings were correlated with a higher Golgi pH and more delayed secretory pathway trafficking in the knock-out, while both processes were less abnormal in the knock-in. Impaired processes are labeled in red, enhanced processes are labeled with green dotted lines. Created in BioRender. Kopp, J. (2024) https://BioRender.com/q94c161

Except α-dystroglycan O-mannosylation and the consequent brain anomalies most cellular and glycosylation alterations described in this report are milder in presence of V0a2-RQ than in the V0a2 knockout and this difference correlates to a less alkaline Golgi pH in *Atp6v0a2*^*RQ/RQ*^ cells (Fig. 7). It is paradoxical that the Golgi pH was less abnormal in presence of V0a2-RQ than in the V0a2 knockout. A possible explanation could be rescuing effects of increased V0a1 protein levels detected in *Atp6v0a2*^*RQ/RQ*^ MEFs. The reason for this is currently unknown and whether the V0a1 subunit resides in the Golgi compartment in the *Atp6v0a2*^*RQ/RQ*^ MEFs can currently not be determined since the available antibodies do not function in immunofluorescence.

Animal models with mutated V0a subunit proton channel arginine residues have been generated for murine V0a3 and for V0a1 in flies [58, 59]. The murine p.(Arg740Ser) V0a3 mutation has an unusual dominant negative effect leading to mild osteopetrosis in heterozygotes. Expression of V0a1 harboring the p.(Arg755Ala) mutation in *drosophila Atp6v0a1* (*vha100*) knockouts rescues neuronal function and intracellular trafficking, but leads to neuronal apoptosis at higher expression levels. Both examples suggest a kind of gain-of-function effect of the arginine-mutated V0a subunits, which cannot be excluded for V0a2-RQ, e.g. as a reason for the stronger neuronal abnormalities in *Atp6v0a2*^*RQ/RQ*^ mutants. Using a yeast strain harboring the corresponding mutation R735Q in vph1p, which functionally corresponds to V0a1 in mammals, Coonrod et al. demonstrated that vacuole fusion is exclusively dependent on proton transport excluding additional functions of vph1p in membrane fusion [20]. Whether V0a2 might have physiologically relevant pH-independent roles in regulating the function and fusion of intracellular compartments will have to be determined in future investigations.

## Materials and methods

### Generation of *Atp6v0a2* mouse models

To generate the targeting vectors for the *Atp6v0a2*^*−/−*^ and *Atp6v0a2*^*RQ/RQ*^ mice a modified recombineering protocol was used [60]. Exons 10 to 20 of the murine *Atp6v0a2* gene (NCBI38: Chr.5 124,711,748–124,723,441) were transferred from a bacmid (BMQ-337012, Sanger resources) to the pDTA vector (kindly provided by Carmen Birchmeier, Max-Delbrück-Center, Berlin) by homologous recombination, resulting in the genomic subclone. The neomycin cassette flanked by FRT and loxP sites was introduced between the exons 14 and 15 by homologous recombination between the pHW025 vector (kindly provided by Carmen Birchmeier, Max-Delbrück-Center, Berlin) and the genomic subclone. Utilizing cre recombinase the neomycin cassette was deleted, resulting in a single loxP site between exon 14 and 15. This vector was digested with EcoRV between Exon 17 and 18 and ligated with a digestion product of the pHW025 derived by EcoR1 and Ale1 and subsequent blunting (Fig. S1A). G4 embryonal stem cells were used for homologous recombination (Andras Nagy, MSHRI, Toronto) and aggregated with donor morulae. The resulting mouse line was crossed with germline cre recombinase expressing mice (Hprt-cre mice) giving rise to the *Atp6v0a2*^*−/−*^ mouse line (Fig. S1B). For the generation of the *Atp6v0a2*^RQ^ targeting vector a PCR product of the vector ranging from the neomycin cassette to the intron 18 was mutated within the exon 18 to generate the R755Q mutation. This PCR-fragment as well as the *Atp6v0a2*^*−/−*^ targeting vector were digested with RsrII and Nde1 and ligated together (Fig. S1**C**). Mating of the resulting mice with germline flippase recombinase expressing mice (CMV-Flip mice) resulted in the *Atp6v0a2*^RQ^ mouse line (Fig. S1D). For genotyping of the mice, PCR was performed as described (Fig. S2).

Resulting heterozygous *Atp6v0a2* knock-out and knock-in mice were inbred to produce homozygous mice (*Atp6v0a2*^*−/−*^*, Atp6v0a2*^*RQ/RQ*^). Experiments were performed with mice in C57Bl/6 background and littermates were used as controls. Mice were housed in the animal facility of the Max Planck Institute for Molecular Genetics. 3R rules were followed in that the minimum numbers of animals were used for experiments and that the maximum number of experiments were performed in vitro. Permissions for animal breeding and experiments were obtained from the Landesamt für Gesundheit und Soziales (LAGeSo) in Berlin, Germany (No. G0346/13, G0176/19), and the Landesamt für Verbraucherschutz und Lebensmittelsicherheit (LAVES) in Oldenburg, Germany (No. 20/3483).

### mRNA expression analysis

Tissue was snap frozen and crushed using a mortar. Total RNA was extracted from homogenized tissue with AllPrep DNA/RNA/Protein Mini Kit (Qiagen, Hilden, Germany). Total cDNA was reverse transcribed by RevertAid H Minus First Strand cDNA Synthesis Kit (ThermoFisher, Waltham, USA). Quantitative PCR was performed using EVAgreen (Solis BioDyne, Tartu, Estonia) on a QuantStudio 3 Real-Time-PCR System (ThermoFisher, Waltham, USA). All primer sequences are available in Table S4.

### Spermiogram analysis

Spermatozoa were freshly isolated from the cauda epididymis of adult mice and 5 min incubated in Human Tubal Fluid medium. Sperm motility and concentration were measured using IVOS sperm analyzer (Hamilton Thorne, Beverly, USA). Sperm were observed for 30 s and images were acquired at a frequency of 60 Hz. The cutoff of motile sperm was 30 μm/s for linear velocity.

### Histology

Mice were deeply anaesthetized and transcardially perfused with 4% PFA and organs were sampled. Tissues for paraffin sectioning were fixed in 4% PFA at 4 °C overnight and sectioned at Microm HM 355 S (Microm, Walldorf, Germany) at 5 µm thickness. Brain samples were stained with kresyl violet. Testis, epididymis and skin samples were stained with hematoxylin/eosin.

### Ultrastructural analysis

Skin samples were fixed for at least 2 h at room temperature in 2.5% glutaraldehyde solution in 0.1 M cacodylate buffer pH 7.4, cut into pieces of ca. 1 mm^3^, washed in buffer, postfixed for 1 h in 1% aqueous osmium tetroxide, rinsed in water, dehydrated through graded ethanol solutions, transferred into propylene oxide, and embedded in epoxy resin (glycidether 100). Semithin and ultrathin sections were cut with an ultramicrotome (Reichert Ultracut E)(Leica Microsystems, Nussloch, Germany). Semithin sections of 1 µm were stained with methylene blue. 60–80 nm ultrathin sections were treated with uranyl acetate and lead citrate and examined with an electron microscope JEM 1400 equipped with a 2K TVIPS CCD Camera.

Testes were collected from adult mice and small pieces were fixed in 1.5% glutaraldehyde, 1.5% formaldehyde, 0.15 mol/L HEPES/KOH (pH 7.4) at 4 °C until embedding and sectioning. For epoxy resin embedding, samples were postfixed in 1% osmium tetroxide in aqua bidest, stained in half-saturated watery uranyl acetate, dehydrated in an ascending ethanol series and finally embedded in Agar 100 (Agar scientific Ltd., Stansted, UK). Ultrathin sections were cut using an ultramicrotome and examined by TEM (Zeiss EM 902, Oberkochen, Germany). Digital images were captured with a slow-scan 2 K CCD camera (TRS, Tröndle, Moorenweis, Germany).

### Cell culture

Mouse embryonic fibroblasts (MEFs) were isolated from e13.5 embryos using trypsin–EDTA (Gibco, ThermoFisher, Waltham, USA) and cultivated in DMEM (Lonza, Basel, Switzerland) supplemented with 10% fetal calf serum (Gibco, ThermoFisher, Waltham, USA), 1% UltraGlutamine (Lonza, Basel, Switzerland) and 1% penicillin/streptomycin (Lonza, Basel, Switzerland). As positive controls, the wildtype cells were treated with monensin (20 µM), 2F-peracetyl-fucose (200 µM), chloroquine (50 µM), bafilomycin A1 (200 µM) or ammonium chloride (25 µM) (all from Sigma Aldrich, St. Louis, Missouri, USA). MEFs were immortalized with SV40 large T antigen transfection (Addgene plasmid #21826) using Lipofectamine 3000 Reagents according to manufactural instruction (ThermoFisher, Waltham, USA).

### Viral overexpression of V0a2

For viral overexpression of the V0a2 subunit, the human *ATP6V0A2* cDNA sequence tagged with V5 (GKPIPNPLLGLDST) was cloned into the vector pLXIN (Clontech Laboratories, California, USA) with or without the R755Q mutation. Using Phoenix ampho packaging cells (ATTC, Manassas, USA), retroviruses were produced to infect HeLa cells. Stable cell lines with mild overexpression were generated by selection with 0.6 mg/ml G418 (Gibco, ThermoFisher, Waltham, USA).

### Immunostaining

Paraffin sections were deparaffinized, rehydrated and equilibrated in PBS. Antigen unmasking was performed using citric acid (pH 6.0) or pepsin antigen retriever (Sigma Aldrich, St. Louis, Missouri, USA) followed by permeabilisation and blocking in 0.2% Triton-X/5% BSA/5% goat or donkey serum. Antibodies used are as follows: Anti-laminin (#L9393, Sigma-Aldrich, St. Louis, USA), Anti-Ctip2 (#ab18465, Abcam, Danaher, Washington, USA), Anti-Tbr2 (#AB15894, MerckMillipore, Darmstadt, Germany), Anti-IIH6 α-Dag1 (#05–593, MerckMillipore, Darmstadt, Germany), Anti-ß-Dag1 (#7D11, Developmental Studies Hybridoma Bank, created by the NICHD of the NIH and maintained at The University of Iowa, Department of Biology, Iowa City, IA 52242); Acrosome were stained with Lectin PNA conjugated with Alexa Fluor 568 (#L32458, Sigma-Aldrich, St. Louis, USA). Cultured cells were fixed with 4% PFA, permeabilized and blocked with 0.1% Saponin/ 3% BSA. The following antibodies were applied: Anti-GM130 (#610823, BD Biosciences, Heidelberg, Germany), anti-Calreticulin (#ab92516, Abcam, Danaher, Washington, USA), antiTGN46 (#AHP500GT, Bio-Rad, Feldkirchen, Germany), anti-GOPC (#12163-1-AP, Proteintech, Planegg-Martinsried, Germany), anti-eGFP (#ab6556, Abcam, Danaher, Washington, USA) and anti-V5 (#V8012, Sigma-Aldrich, St.Louis, USA). Sections and cells were decorated with secondary anti-IgG antibodies conjugated with Alexa Fluor 488 (#A21202, Invitrogen, ThermoFisher, Waltham, USA) and Alexa Flour 555 (#A21572, Invitrogen, ThermoFisher, Waltham, USA) or primary antibody was direct labeled with APEX antibody labeling kit (ThermoFisher, Waltham, USA). Fucose residues were stained with Lectin AAL (#B-1395, Vector Laboratories, Newark, USA) for 1 h and detected with streptavidin Alexa Fluor 555 conjugate (#S21381, Invitrogen, ThermoFisher, Waltham, USA). DNA was stained by DAPI (#D1306, Invitrogen, ThermoFisher, Waltham, USA). Sections and cells were mounted in FluoromountG (SouthernBiotech, Birmingham, USA). Fluorescence pictures were taken with LSM 700 (Zeiss, Jena, Germany), Celldiscoverer 7 (Zeiss, Jena, Germany) or BX60 microscope (Olympus, Tokyo, Japan). To facilitate detailed analyses of co-localization, separation of Golgi stacks was induced through treatment of cells for 3 h with 33 µM nocodazol (Sigma-Aldrich, St. Louis, USA).

### Metabolic labeling of sialylated glycoproteins using ManAz

Cells were seeded on coverslips and preincubated with 50 µM Chloroquine (CQ), 200 mM Bafilomycin a1 (Baf), 2 µM Tunicamycin (TN). The next day, cells were pulsed 6 h with 50 µM tetraacetylated N-Azidoacetyl-mannosamine (ManNAz, Jena Bioscience, Jena, Germany). Cells were washed with PBS, fixed with 4% PFA and permeabilized 15 min with 0.5% Triton-X. Cells were labelled with click reaction solution (10 µM Fluor 488-Alkyne fluorescent probe/150 µM CuSO_4_5H_2_O/300 µM BTTAA/2.5 mM Ascorbic acid/100 mM K_2_HPO_4_) for 1 h and nuclei were stained by DAPI. The fluorescence intensities of Golgi apparatus from individual cells were measured using Operetta High Content Imaging System (PerkinElmer, Rodgau, Germany). The experiment was performed three times.

### Measurement of trans Golgi pH

The constructs coding for RpHLuorin2 fused to truncated GalT and GPI-anchored RpHLuorin2 have been described [27] and are deposited at Addgene (plasmid 171,719 and 171,721 respectively). MEF cells were maintained in high glucose DMEM supplemented with 10% fetal bovine serum (FBS) and 1% antibiotic–antimycotic solution (15240-062; Gibco, ThermoFisher, Waltham, USA). Cells were tested for mycoplasma contamination. MEF cells were transfected by electroporation with the plasmids using the NEON Transfection System (Invitrogen, ThermoFisher, Waltham, USA) with 10 μl NeonTips (MPK1025; ThermoFisher, Waltham, USA) using 1 pulse of 1350 V and 30 ms. The concentration of DNA used was 0.5 μg per 1 million cells. Cells were imaged 48 h post-transfection. Only cells expressing low to moderate levels of the transfected plasmids, based on fluorescence intensity and manual localization assessment, were chosen for subsequent microscopic analyses.

Imaging of cells took place in DMEM lacking phenol red, except for the pH calibration curve which was obtained in pH buffers composed of 125 mM KCl, 25 mM NaCl, and 25 mM N-[2-hydroxyethyl]-piperazine-N-[2-ethanesulfonic acid] (HEPES, pH 7.5, 7.0 and pH 8.0) or 25 mM 2-[N-morpholino] ethanesulfonic acid (MES, pH 6.5, 6.0, 5.5, 5.0 or 4.5). Each buffer solution was adjusted to the final pH using 1 M NaOH or 1 M HCl at 37 °C. For calibration with GPI-RpHLuorin2, the cells were preincubated in pH buffer for 15 min at 37 °C.

For microscopy, 50,000 MEF cells were seeded in 100 μl DMEM lacking phenol red and FBS in a four-compartment glass-bottom dish (627,870; Greiner, Kremsmünster, Austria). 4 h after transfection, the cell medium was refreshed with new DMEM medium containing FBS and cells were cultured overnight at 37 °C and 5% CO_2_. 48 h after transfection the medium was changed with imaging medium (DMEM without phenol red) 1 h before imaging. For the Bafilomycin A1 conditions, cells were treated with 200 nM Bafilomycin A1 (cat.code tlrl-baf1; InvivoGene) in DMSO (1st experiment) or 100 nM Bafilomycin A1 with 20 μM carbonyl cyanide m-chlorophenyl hydrazone (CCCP; second and third experiment) for 1 h prior to live imaging. Imaging was performed at 37 °C using a LSM800 Zeiss confocal laser scanning microscope with a 40 × 0.95 NA water immersion objective, using sequential excitation at 405 and 488 nm (Zeiss, Jena, Germany). Images were analyzed using FIJI-ImageJ as described [27].

### Brefeldin A-induced golgi collapse

Cells were seeded on coverslips. The next day, cells were incubated with 5 µg/ml Brefeldin A (BFA) for 8 min and fixed in 4% PFA for 10 min. Cells were washed three times in PBS, permeabilized and blocked with 0.1% saponin in 3% BSA. Immunofluorescence staining was performed for Gm130 and Calreticulin. Pictures were taken with microscope BX60 (Olympus, Tokyo, Japan) and LSM 700 (Zeiss, Jena, Germany). At least 100 cells per sample were counted and the experiment was performed twice.

### RUSH assay

Immortalized MEFs were seeded on coverslips. The next day, the cells were transfected with a Str-KDEL-IRES-TNFa-GFP-RUSH construct (according to [25, 61]) using Lipofectamine 3000 Reagents according to manufactural instruction (ThermoFisher, Waltham, USA). 24 h post transfection cells were incubated with 40 µM biotin (Roth, Karlsruhe, Germany) for 10, 15, 20 and 25 min and fixed in 4% PFA for 10 min. Cells were washed three times in PBS, permeabilized and blocked with 0.1% saponin in 3% BSA. Immunofluorescence staining was performed for GM130 and eGFP. Pictures were taken with Celldiscoverer 7 (Zeiss, Jena, Germany) and LSM 700 (Zeiss, Jena, Germany). At least 100 transfected cells per sample and timepoint were analyzed.

### Flow cytometry analysis

Flow cytometry analysis was used to quantify an Aleuria Aurantia Lectin (AAL) surface staining on MEFs. Measurements were performed on a Cytoflex LX device (Beckman Coulter Genomics, Brea, USA) using 96-well U-bottom plates for the measurement. For the quantification, approximately 100,000 cells were transferred onto the 96-U-bottom-well plate and washed by adding 200 μL of PBS, centrifuging the plates at 400 g for 5 min at RT, discarding the supernatants, and resuspending the pellets in the remaining volume by vortexing briefly. For the individual staining procedure, a mastermix of the fluorophore conjugated lectin diluted in PBS was prepared. Twenty microliters of the mastermix were added per well. The plates were incubated for 15 min at 4 °C. The first staining step with Aleuria Aurantia Lectin (AAL), Fluorescein (FL-1391-1) (Vector Laboratories, Newark, USA) was performed with dilution (1:100). After incubation for 15 min, one washing step was performed prior to staining with DAPI. DAPI was diluted in PBS (1:100,000) before acquiring 50 µL. At least 600 cells/condition were analyzed. Data were analyzed using FlowJo_V10.

### Membrane precipitation

Cells were rinsed with ice‐cold PBS, resuspended in homogenization buffer (20 mM HEPES (pH 7.5), 125 mM KCl, 50 mM sucrose, 1 mM EDTA, protease and phosphatase inhibitors), and homogenized by 12 strokes with a KIMBLE Dounce tissue grinder with a large clearance pestle (SigmaAldrich, St. Louis, USA). Then, nuclei were removed by centrifugation at 800 × g for 5 min and membranes pelleted from post-nuclear supernatants by centrifugation at 18,000 × g for 20 min. Resulting pellets were resuspended in lysis buffer (50 mM HEPES pH 7.4, 40 mM NaCl, 2 mM EDTA, 1 mM sodium orthovanadate, 50 mM sodium fluoride, 10 mM sodium pyrophosphate, 10 mM sodium glycerophosphate, 1% Triton X‐100, 1 × Halt protease and phosphatase inhibitor cocktails).

### Immunoblotting

Proteins were extracted with AllPrep DNA/RNA/Protein Mini Kit (Qiagen, Hilden, Germany) and solved in 5% SDS RIPA buffer (150 mM NaCl, 50 mM Tris, 5 mM EDTA, 1% Triton X-100, 0.25% Desoxycholate, 5% SDS) supplemented with protease inhibitor (Complete, Roche, Mannheim, Germany). 15 to 20 μg of protein per lane were separated by SDS-PAGE and subsequently transferred to nitrocellulose membrane. After semidry blotting membranes were blocked 1 h with Blocking buffer (Li-Cor Biosciences, Lincoln, USA) and probed with primary antibodies. Antibodies were used as follows: Anti-Atp6v0a1 (#13828-1-AP, Proteintech, Planegg-Martinsried, Germany), Anti-Atp6v0a2 (#ab96803, Abcam, Danaher, Washington, USA), Anti-Atp6v0d1 (#ab202899, Abcam, Danaher, Washington, USA), Anti-Atp6v1a (#ab199326, Abcam, Danaher, Washington, USA), Anti-Atp6v1b2 (#14617S, Cell Signaling, Danvers, USA), Anti-Eln, Elastin Products Company), Anti-Dcn (#LF-114, Kerafast, Shirley, USA), Anti-Tgn38 (#ab16059, Abcam, Danaher, Washington, USA), Anti-Gpr56 (#MABN310, MerckMillipore, Darmstadt, Germany), Anti-Col3a (#C2C3, GeneTex, Irvine, USA), Anti-Gopc (#ab109119, Abcam, Danaher, Washington, USA), Anti-Gapdh (#AM4300, ThermoFisher, Waltham, USA) and Anti-ActB (#4970, Cell signaling, Danvers, USA). Lectins were produced by Vector Laboratories (Newark, USA): SNA (#B-1305), PNA (#B-1075), E-Pha (#B-1125-2), AAL (#B-1395). Membranes were washed and incubated with IRDye- HRPconjugated secondary antibodies. Lectins were incubated with IRDye-streptavidin. Total protein was stained using Revert™ 700 Total Protein Stain (LI-COR Biosciences, Bad Homburg, Germany). Signals were detected with OdysseyFc Imaging System and densitometric quantification was performed using Image Studio (LI-COR Biosciences, Bad Homburg, Germany). V-ATPase western blots were quantified using Image Lab software (Bio-Rad, Feldkirchen, Germany).

### Proteome analysis

Confluent MEFs were washed twice with HBSS and once with DMEM w/o phenol red (Lonza, Basel, Switzerland). 6 mL DMEM w/o phenol red without FCS were added. After 24 h the supernatants were collected. The remaining cell layers were lysed in lysis buffer (3 M guanidinium chloride/5 mM Tris(2 carboxyethyl)phosphin, 20 mM chloroacetamide und 50 mM Tris(hydroxymethyl)aminomethan pH 8,5) and incubated at 96 °C for 10 min. Protein lysates were analyzed by LC–MS/MS and label-free quantification (LFQ) as previously described [62]. Data analysis was done with Perseus (v1.6.10.43). LFQ intensities, originating from at least two different peptides per protein group were transformed by log2. Proteins differentially expressed by at least one Log2 unit are given in Tables S1 and S2.

### N-Glycan analysis

The proteins/glycoproteins were reduced and carboxyamidomethylated followed by sequential tryptic and peptide N-glycosidase F digestion and Sep-Pak purification. Permethylation of the freeze-dried glycans and MALDI-TOF-MS of permethylated glycans were performed as described elsewhere [63]. Three animals per genotype were analyzed.

### Statistical analysis

One- and two-way analysis of variance (ANOVA), student’s t-test as well as Kruskal–Wallis test with appropriate multiple testing correction were performed using GraphPad prism 8 (v.8.3.0). Animal experiments were repeated at a minimum of three times.

## Supplementary Information

Below is the link to the electronic supplementary material.

Supplementary file1 (XLSX 139 KB)

Supplementary file2 (XLSX 125 KB)

Supplementary file3 (XLSX 11 KB)

Supplementary file4 (XLSX 11 KB)

Supplementary file5 (PPTX 2858 KB)

## Data Availability

The proteomics datasets generated during the current study are not publicly available due to their use in a parallel investigation but are available from the corresponding author on reasonable request and will be released after the publication of the ongoing research effort.
