# Supplementary material for: Golgi pH elevation due to loss of V-ATPase subunit V0a2 function correlates with tissue-specific glycosylation changes and globozoospermia
Source: Cell Mol Life Sci. 2024 Dec 16;82(1):4. doi: 10.1007/s00018-024-05506-7 (PMC11649611; doi:10.1007/s00018-024-05506-7)
Supplement: Supplementary file 5 — Supplementary file5 (PPTX 2858 KB) [file 18_2024_5506_MOESM5_ESM.pptx]

## Slide 1
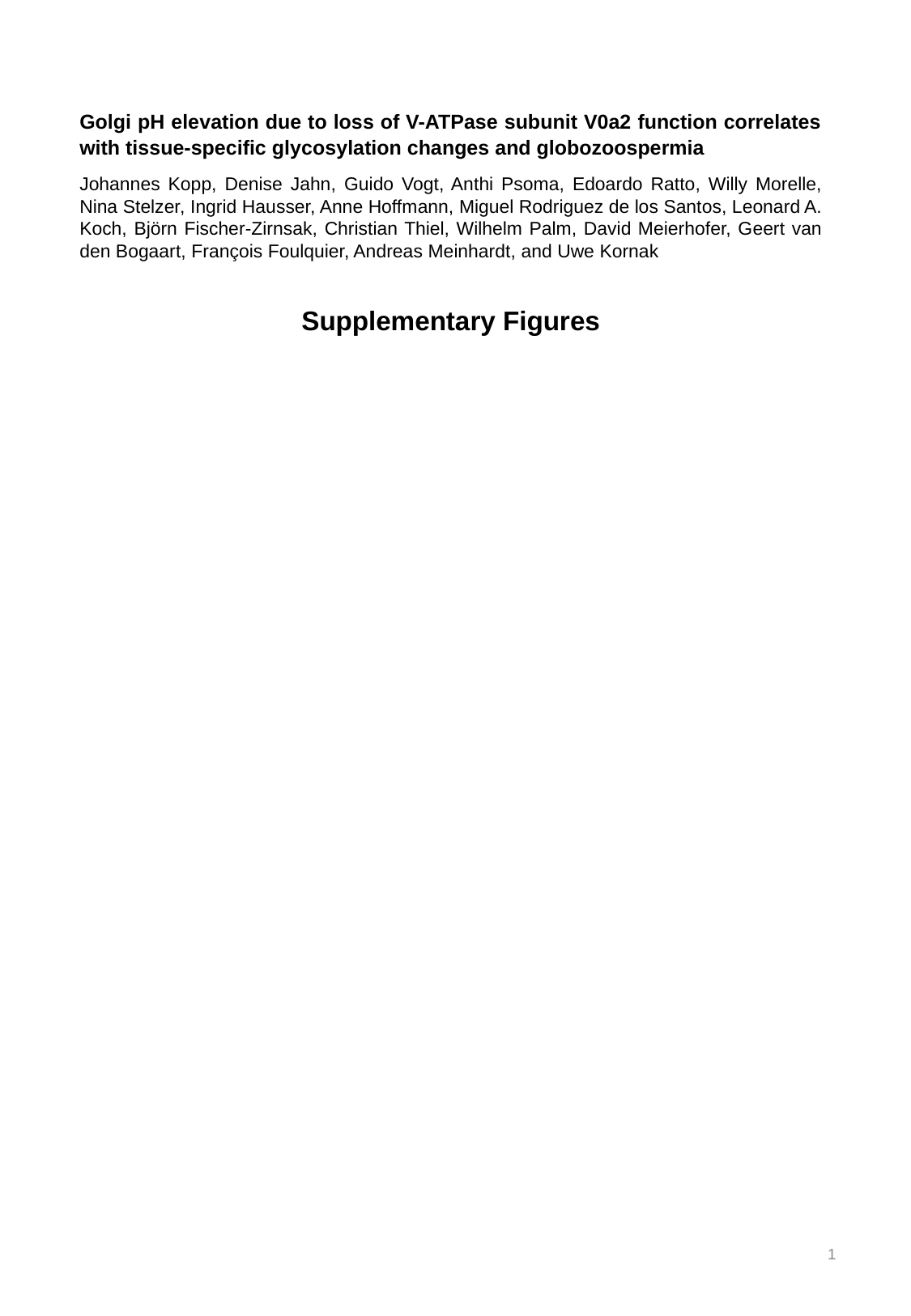

Golgi pH elevation due to loss of V-ATPase subunit V0a2 function correlates with tissue-specific glycosylation changes and globozoospermia
Johannes Kopp, Denise Jahn, Guido Vogt, Anthi Psoma, Edoardo Ratto, Willy Morelle, Nina Stelzer, Ingrid Hausser, Anne Hoffmann, Miguel Rodriguez de los Santos, Leonard A. Koch, Björn Fischer-Zirnsak, Christian Thiel, Wilhelm Palm, David Meierhofer, Geert van den Bogaart, François Foulquier, Andreas Meinhardt, and Uwe Kornak
Supplementary Figures
1

## Slide 2
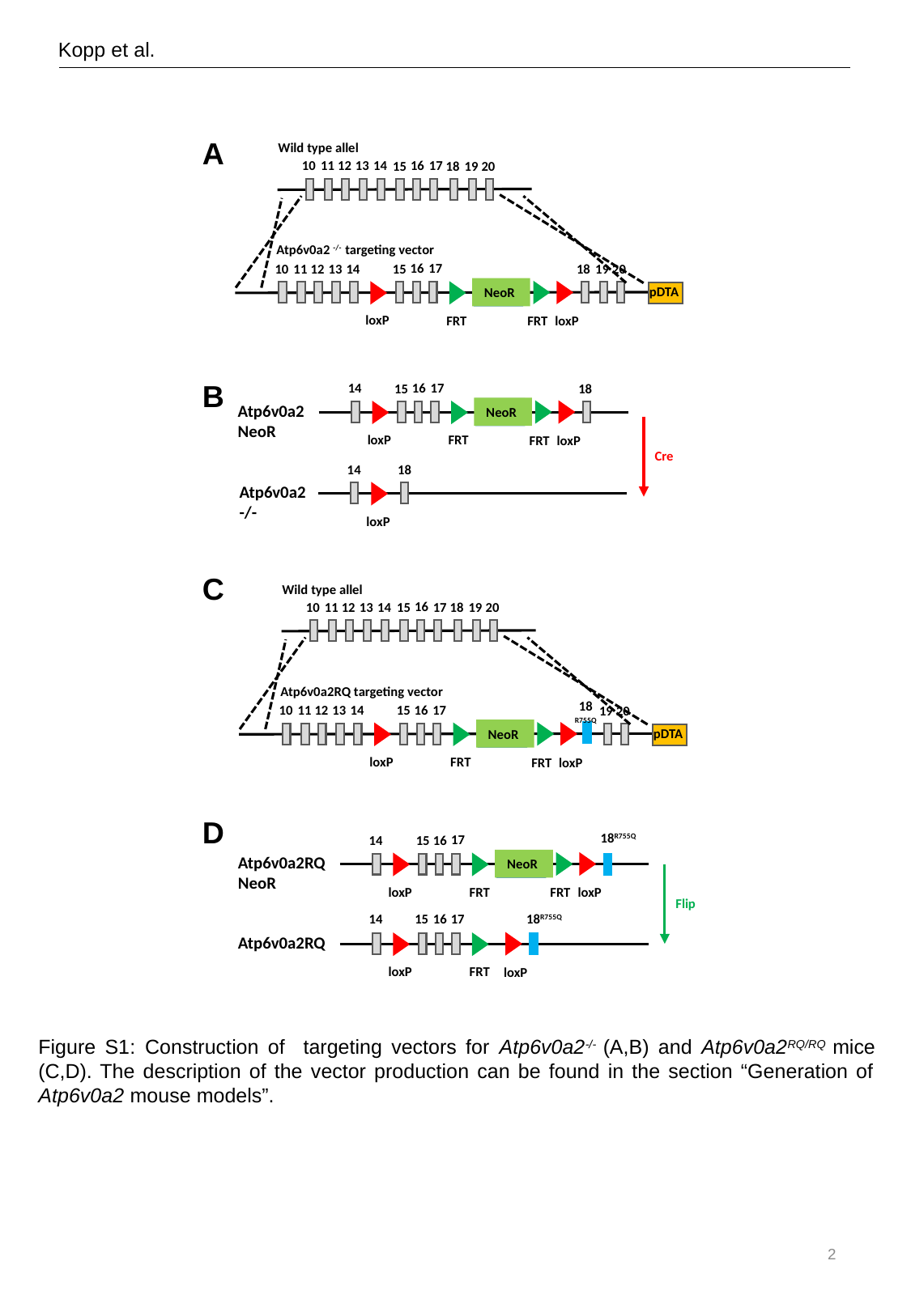

Kopp et al.
A
Wild type allel
16
17
10
11
12
13
14
15
18
19
20
Atp6v0a2 -/- targeting vector
16
17
10
11
12
13
14
15
18
19
20
pDTA
NeoR
loxP
FRT
FRT
loxP
B
16
17
14
15
18
Atp6v0a2
NeoR
NeoR
loxP
FRT
FRT
loxP
Cre
14
18
Atp6v0a2
-/-
loxP
C
Wild type allel
16
17
10
11
12
13
14
15
18
19
20
Atp6v0a2RQ targeting vector
18
R755Q
16
17
10
11
12
13
14
15
19
20
pDTA
NeoR
loxP
FRT
FRT
loxP
D
18R755Q
17
15
16
14
Atp6v0a2RQ
NeoR
NeoR
loxP
FRT
FRT
loxP
Flip
17
16
14
15
18R755Q
Atp6v0a2RQ
loxP
FRT
loxP
Figure S1: Construction of targeting vectors for Atp6v0a2-/- (A,B) and Atp6v0a2RQ/RQ mice (C,D). The description of the vector production can be found in the section “Generation of Atp6v0a2 mouse models”.
2

## Slide 3
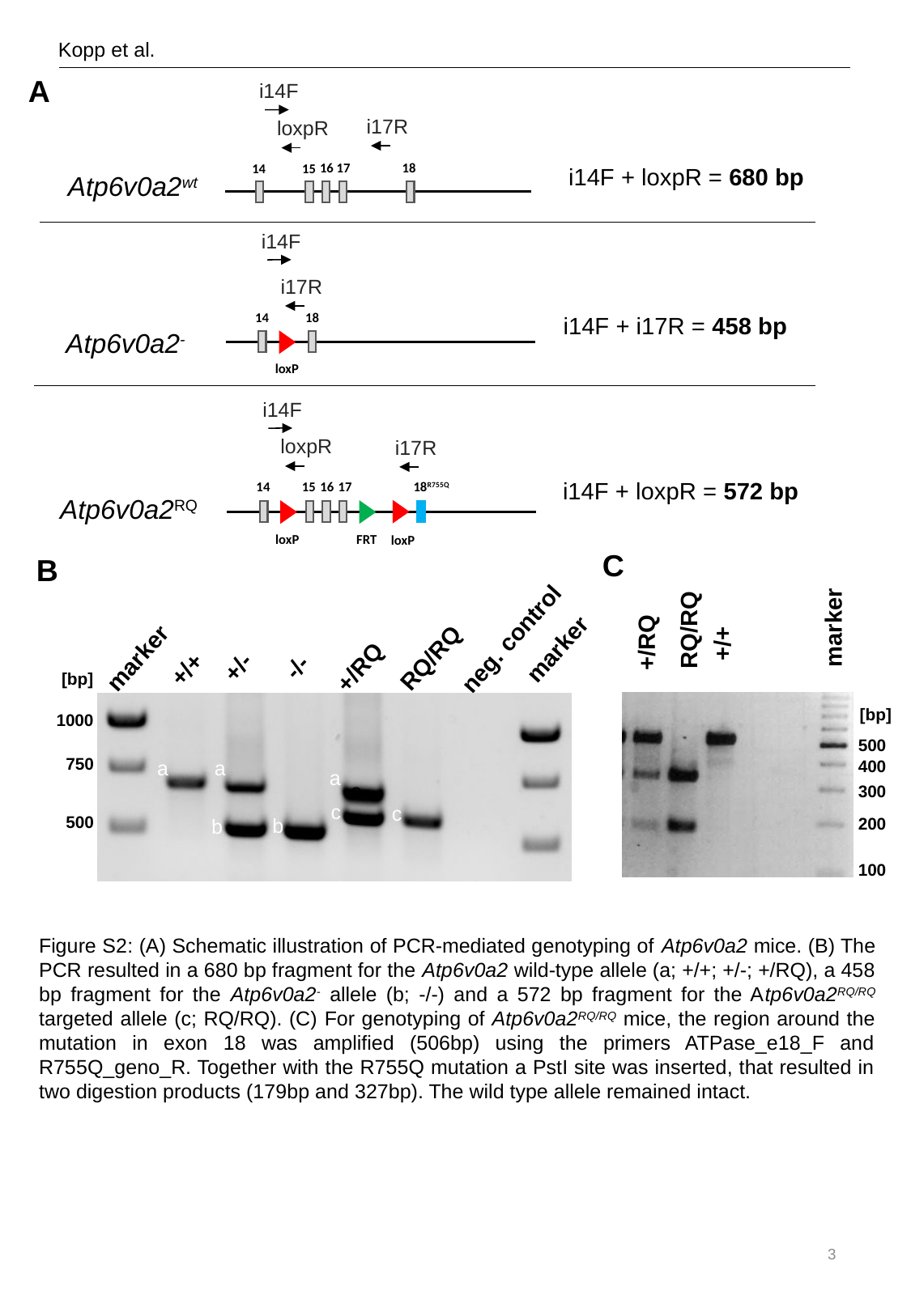

Kopp et al.
A
i14F
i17R
loxpR
16
18
17
14
i14F + loxpR = 680 bp
15
Atp6v0a2wt
i14F
i17R
14
18
i14F + i17R = 458 bp
Atp6v0a2-
loxP
i14F
loxpR
i17R
i14F + loxpR = 572 bp
17
16
14
15
18R755Q
Atp6v0a2RQ
loxP
FRT
loxP
C
B
marker
marker
RQ/RQ
+/-
-/-
+/RQ
+/+
1000
750
a
a
a
a
c
c
500
b
b
neg. control
marker
RQ/RQ
+/RQ
+/+
[bp]
[bp]
500
400
300
200
100
Figure S2: (A) Schematic illustration of PCR-mediated genotyping of Atp6v0a2 mice. (B) The PCR resulted in a 680 bp fragment for the Atp6v0a2 wild-type allele (a; +/+; +/-; +/RQ), a 458 bp fragment for the Atp6v0a2- allele (b; -/-) and a 572 bp fragment for the Atp6v0a2RQ/RQ targeted allele (c; RQ/RQ). (C) For genotyping of Atp6v0a2RQ/RQ mice, the region around the mutation in exon 18 was amplified (506bp) using the primers ATPase_e18_F and R755Q_geno_R. Together with the R755Q mutation a PstI site was inserted, that resulted in two digestion products (179bp and 327bp). The wild type allele remained intact.
3

## Slide 4
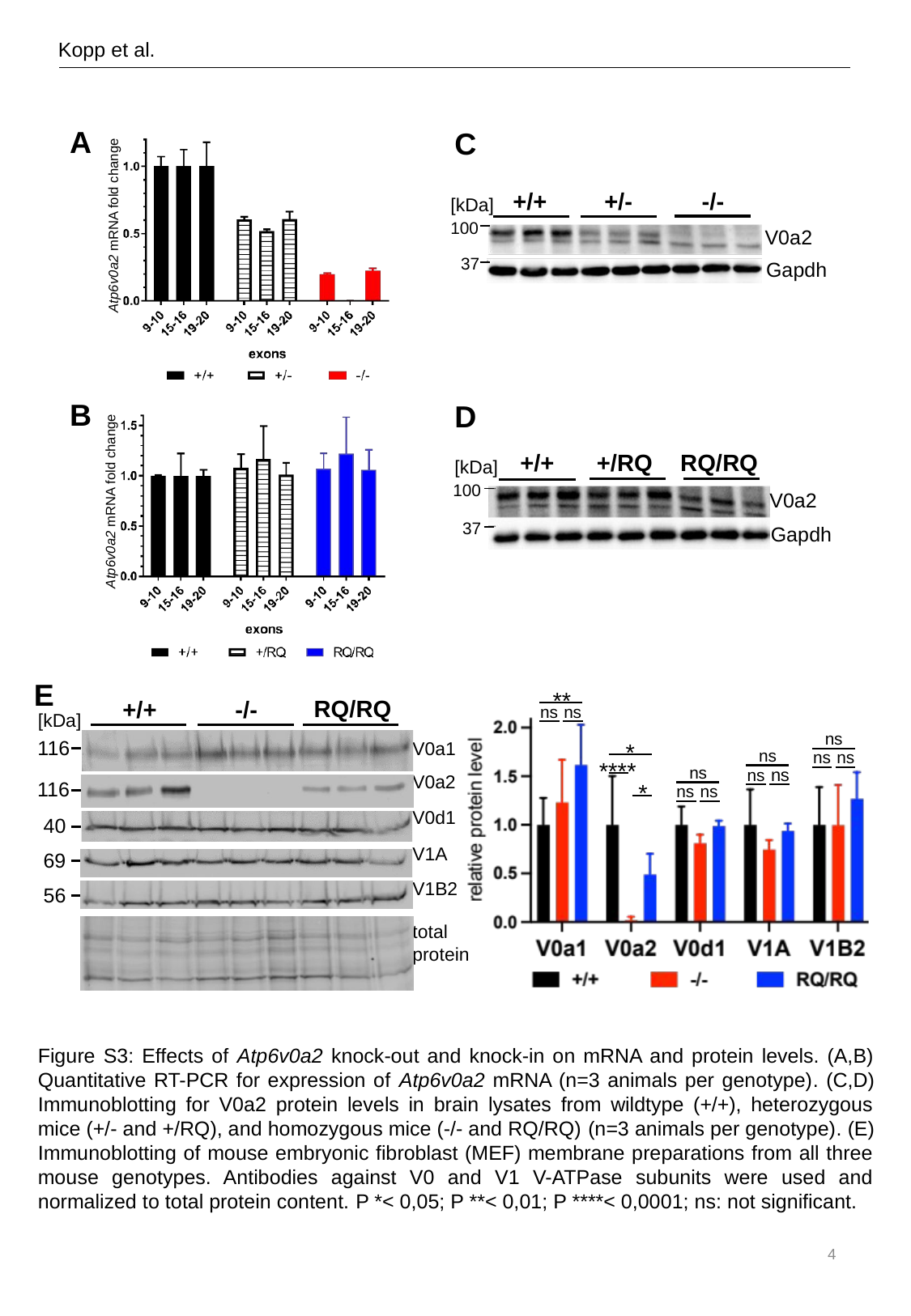

Kopp et al.
A
C
+/-
-/-
+/+
[kDa]
Atp6v0a2 mRNA fold change
100
V0a2
37
Gapdh
B
D
+/+
+/RQ
RQ/RQ
[kDa]
100
V0a2
Atp6v0a2 mRNA fold change
37
Gapdh
E
**
RQ/RQ
-/-
+/+
ns
ns
[kDa]
ns
116
V0a1
*
ns
ns
ns
****
ns
ns
ns
V0a2
116
*
ns
ns
V0d1
40
V1A
69
V1B2
56
total protein
Figure S3: Effects of Atp6v0a2 knock-out and knock-in on mRNA and protein levels. (A,B) Quantitative RT-PCR for expression of Atp6v0a2 mRNA (n=3 animals per genotype). (C,D) Immunoblotting for V0a2 protein levels in brain lysates from wildtype (+/+), heterozygous mice (+/- and +/RQ), and homozygous mice (-/- and RQ/RQ) (n=3 animals per genotype). (E) Immunoblotting of mouse embryonic fibroblast (MEF) membrane preparations from all three mouse genotypes. Antibodies against V0 and V1 V-ATPase subunits were used and normalized to total protein content. P *< 0,05; P **< 0,01; P ****< 0,0001; ns: not significant.
4

## Slide 5
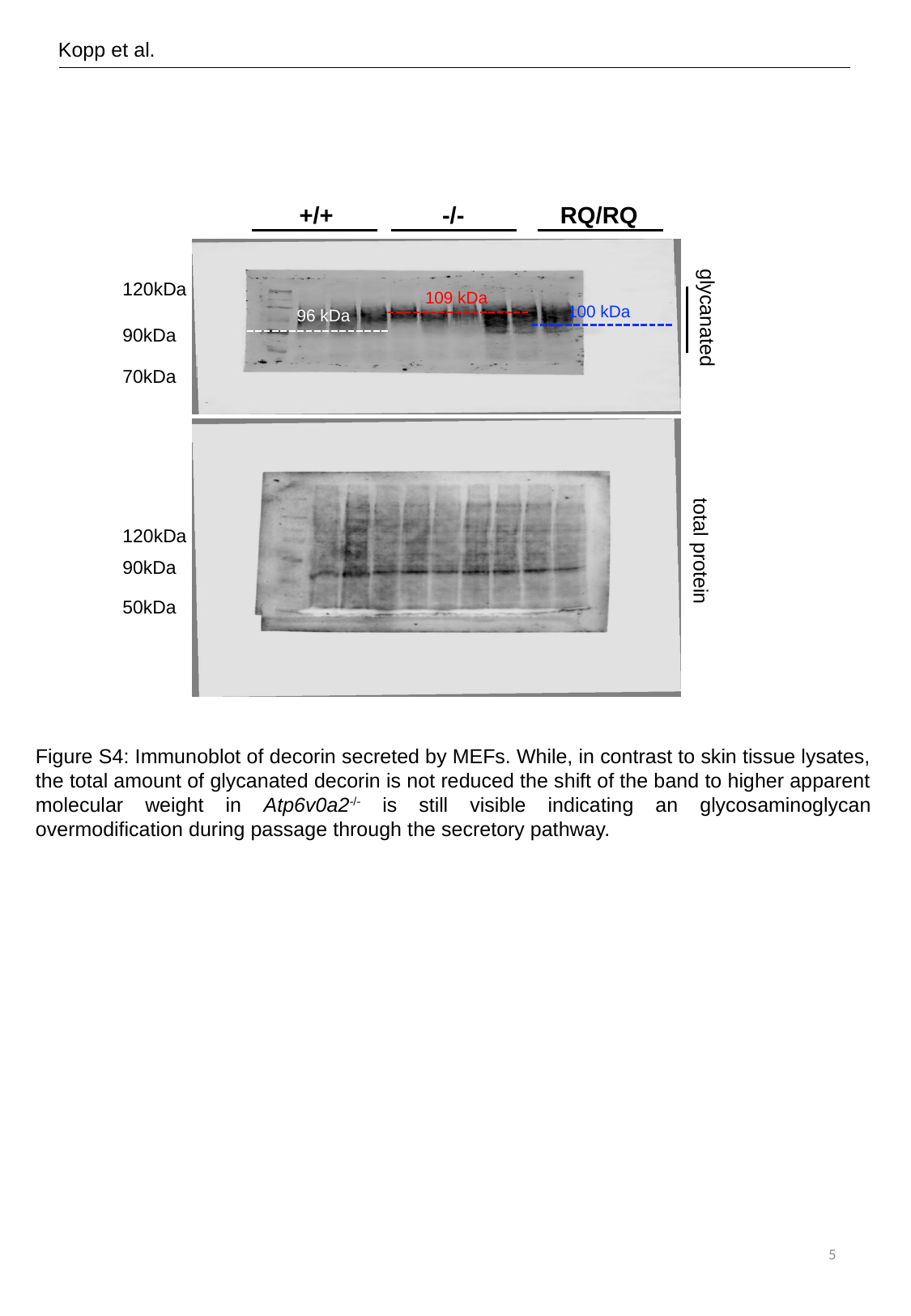

Kopp et al.
+/+
-/-
RQ/RQ
109 kDa
100 kDa
96 kDa
120kDa
glycanated
90kDa
70kDa
120kDa
total protein
90kDa
50kDa
Figure S4: Immunoblot of decorin secreted by MEFs. While, in contrast to skin tissue lysates, the total amount of glycanated decorin is not reduced the shift of the band to higher apparent molecular weight in Atp6v0a2-/- is still visible indicating an glycosaminoglycan overmodification during passage through the secretory pathway.
5

## Slide 6
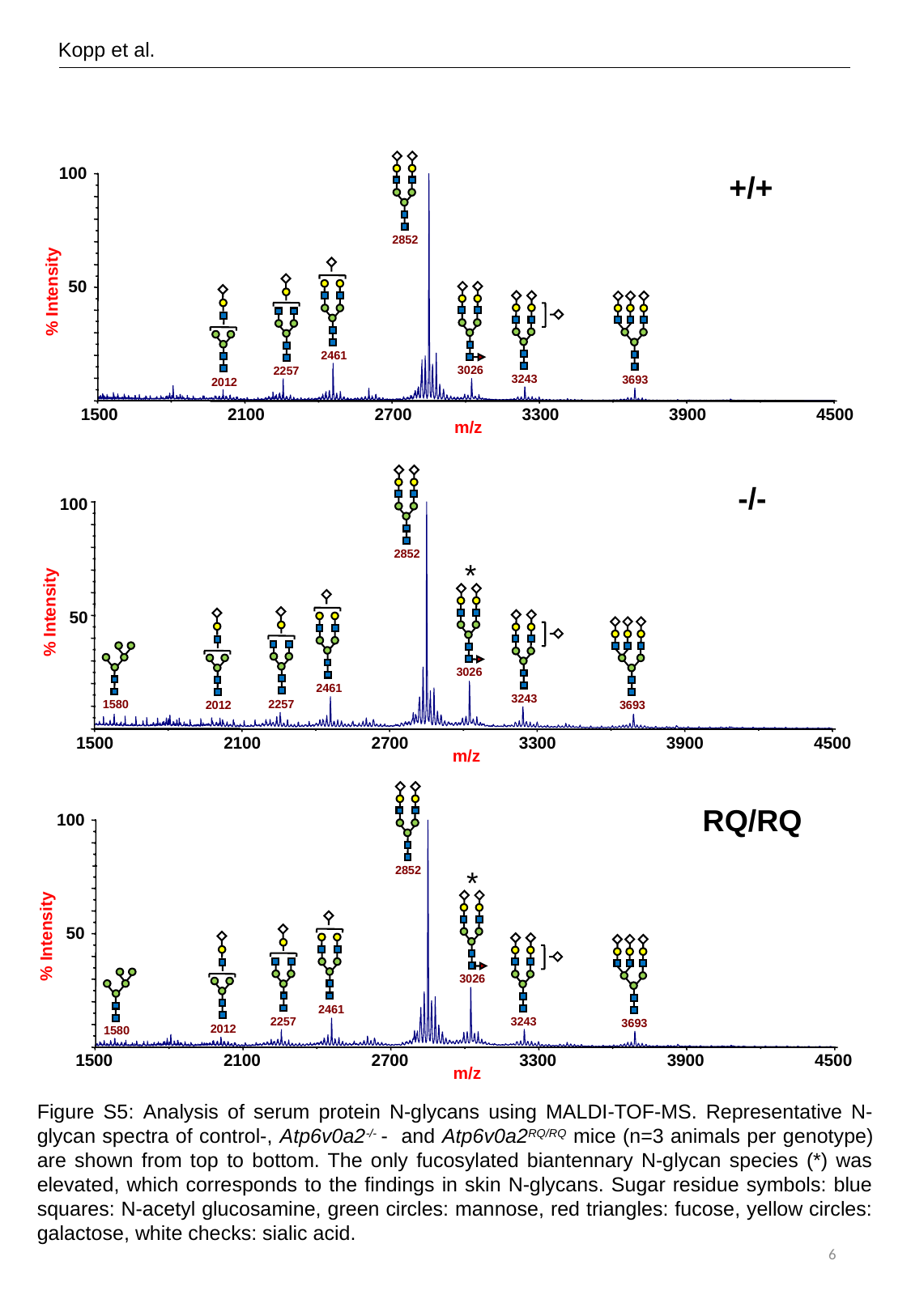

Kopp et al.
2852
+/+
100
2461
2257
50
% Intensity
3026
2012
3243
3693
1500
2100
2700
3300
3900
4500
m/z
2852
-/-
100
*
3026
2461
% Intensity
50
2257
2012
3243
3693
1580
1500
2100
2700
3300
3900
4500
m/z
2852
RQ/RQ
100
*
3026
2461
50
2257
% Intensity
2012
3243
3693
1580
1500
2100
2700
3300
3900
4500
m/z
Figure S5: Analysis of serum protein N-glycans using MALDI-TOF-MS. Representative N-glycan spectra of control-, Atp6v0a2-/- - and Atp6v0a2RQ/RQ mice (n=3 animals per genotype) are shown from top to bottom. The only fucosylated biantennary N-glycan species (*) was elevated, which corresponds to the findings in skin N-glycans. Sugar residue symbols: blue squares: N-acetyl glucosamine, green circles: mannose, red triangles: fucose, yellow circles: galactose, white checks: sialic acid.
6

## Slide 7
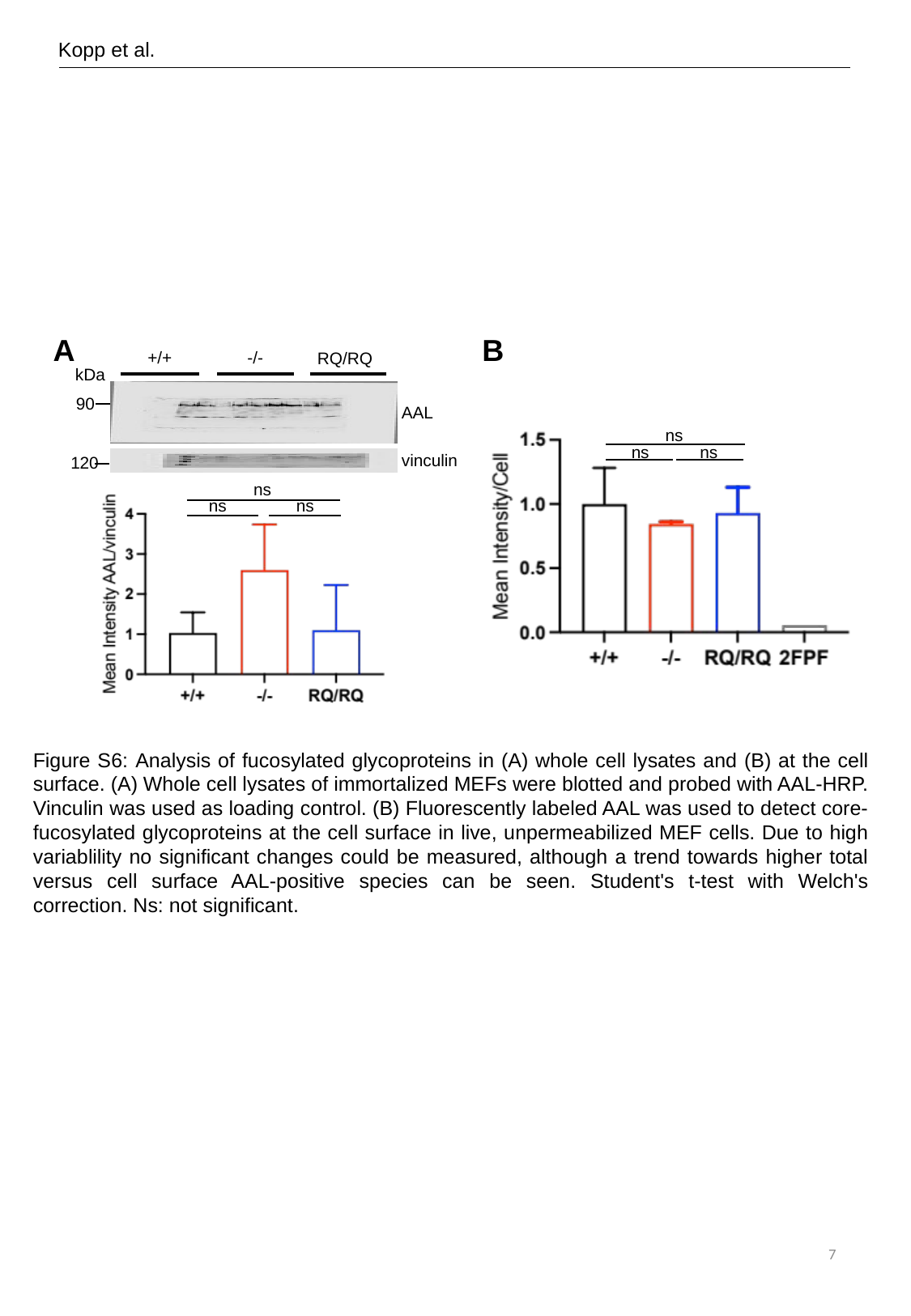

Kopp et al.
A
B
-/-
+/+
RQ/RQ
kDa
90
AAL
ns
ns
ns
vinculin
120
ns
ns
ns
Figure S6: Analysis of fucosylated glycoproteins in (A) whole cell lysates and (B) at the cell surface. (A) Whole cell lysates of immortalized MEFs were blotted and probed with AAL-HRP. Vinculin was used as loading control. (B) Fluorescently labeled AAL was used to detect core-fucosylated glycoproteins at the cell surface in live, unpermeabilized MEF cells. Due to high variablility no significant changes could be measured, although a trend towards higher total versus cell surface AAL-positive species can be seen. Student's t-test with Welch's correction. Ns: not significant.
7

## Slide 8
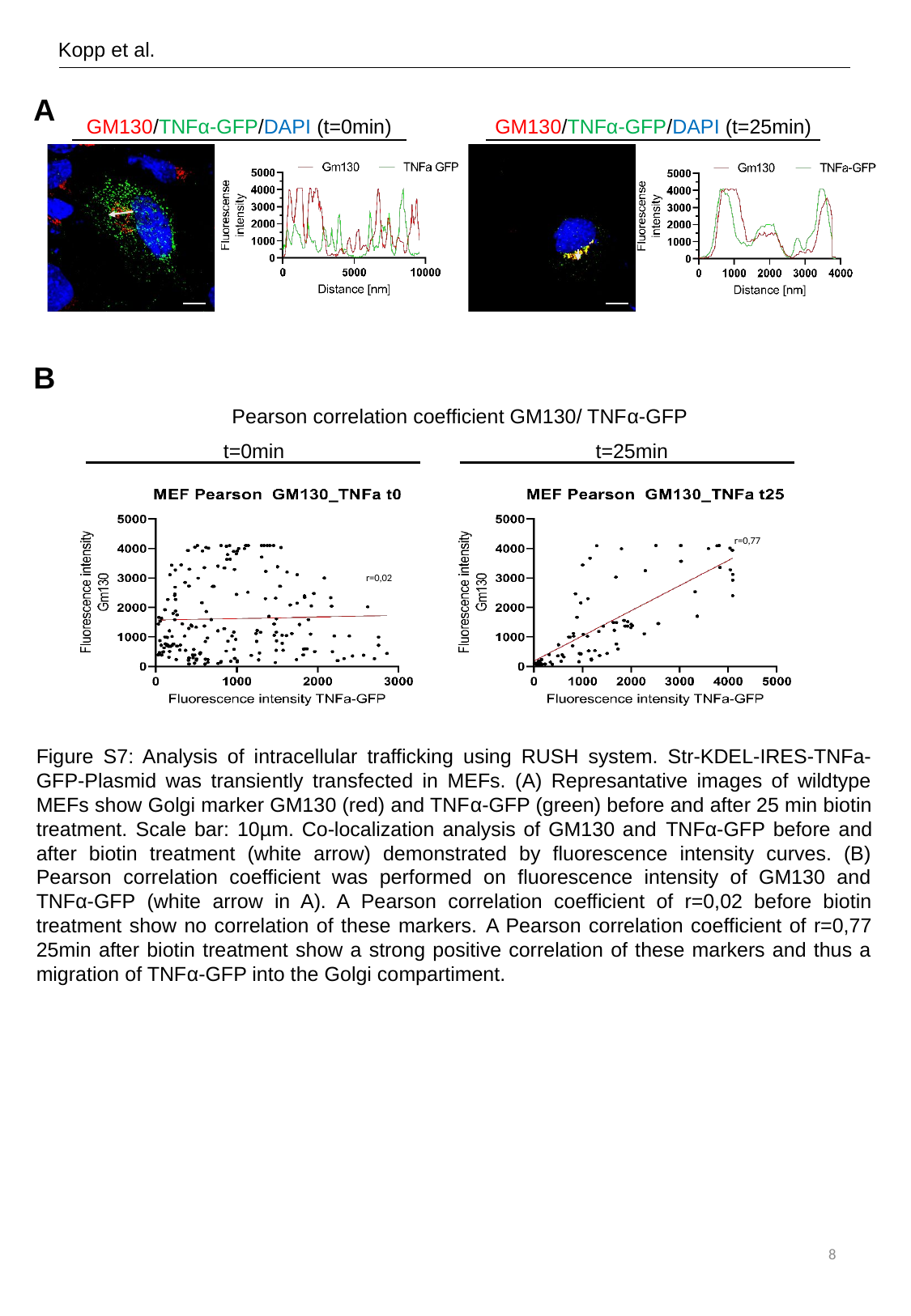

Kopp et al.
A
GM130/TNFα-GFP/DAPI (t=25min)
GM130/TNFα-GFP/DAPI (t=0min)
B
Pearson correlation coefficient GM130/ TNFα-GFP
t=0min
t=25min
r=0,77
r=0,02
Figure S7: Analysis of intracellular trafficking using RUSH system. Str-KDEL-IRES-TNFa-GFP-Plasmid was transiently transfected in MEFs. (A) Represantative images of wildtype MEFs show Golgi marker GM130 (red) and TNFα-GFP (green) before and after 25 min biotin treatment. Scale bar: 10µm. Co-localization analysis of GM130 and TNFα-GFP before and after biotin treatment (white arrow) demonstrated by fluorescence intensity curves. (B) Pearson correlation coefficient was performed on fluorescence intensity of GM130 and TNFα-GFP (white arrow in A). A Pearson correlation coefficient of r=0,02 before biotin treatment show no correlation of these markers. A Pearson correlation coefficient of r=0,77 25min after biotin treatment show a strong positive correlation of these markers and thus a migration of TNFα-GFP into the Golgi compartiment.
8

## Slide 9
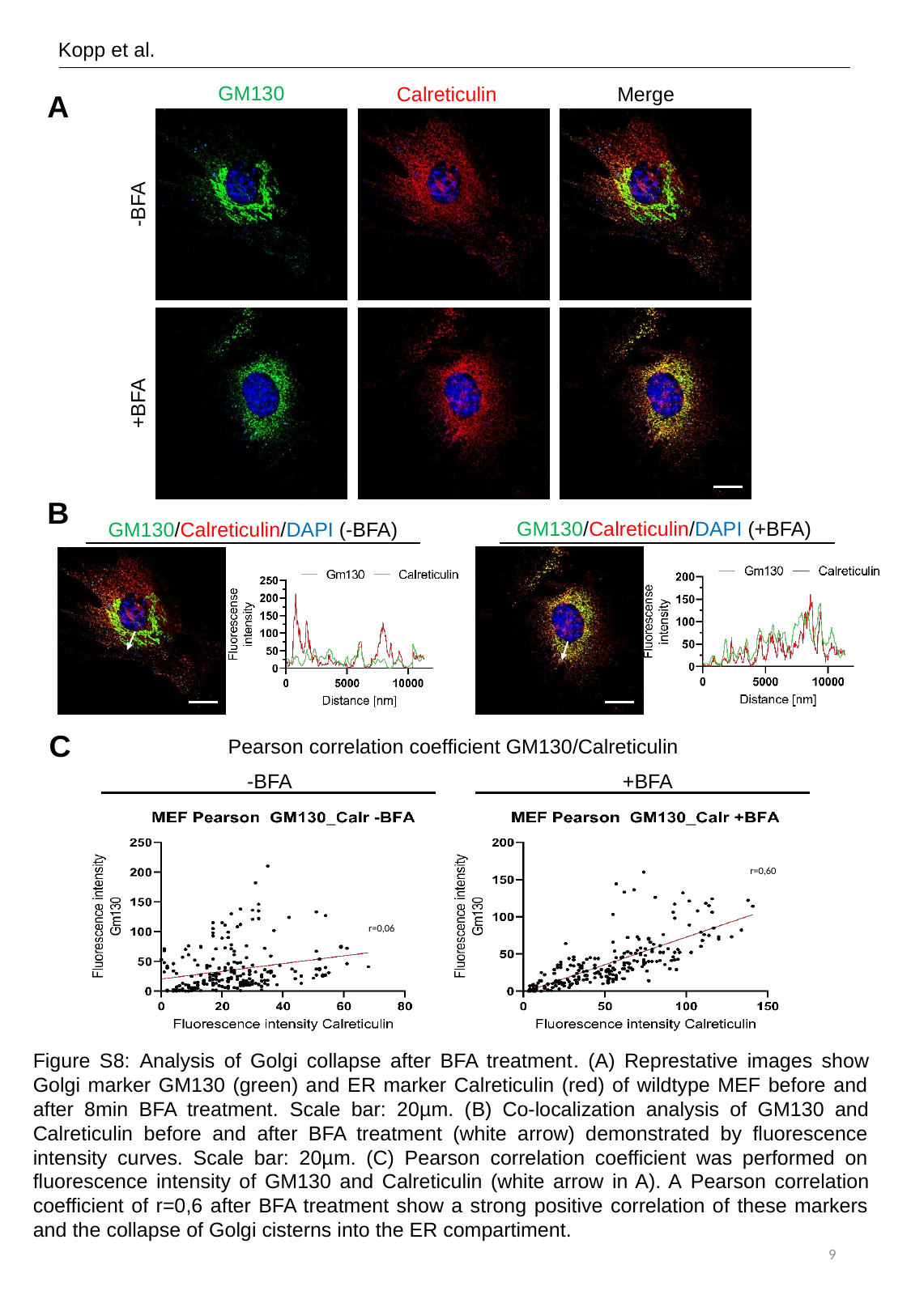

Kopp et al.
GM130
Calreticulin
Merge
A
-BFA
+BFA
B
GM130/Calreticulin/DAPI (+BFA)
GM130/Calreticulin/DAPI (-BFA)
C
Pearson correlation coefficient GM130/Calreticulin
-BFA
+BFA
r=0,60
r=0,06
Figure S8: Analysis of Golgi collapse after BFA treatment. (A) Represtative images show Golgi marker GM130 (green) and ER marker Calreticulin (red) of wildtype MEF before and after 8min BFA treatment. Scale bar: 20µm. (B) Co-localization analysis of GM130 and Calreticulin before and after BFA treatment (white arrow) demonstrated by fluorescence intensity curves. Scale bar: 20µm. (C) Pearson correlation coefficient was performed on fluorescence intensity of GM130 and Calreticulin (white arrow in A). A Pearson correlation coefficient of r=0,6 after BFA treatment show a strong positive correlation of these markers and the collapse of Golgi cisterns into the ER compartiment.
9

## Slide 10
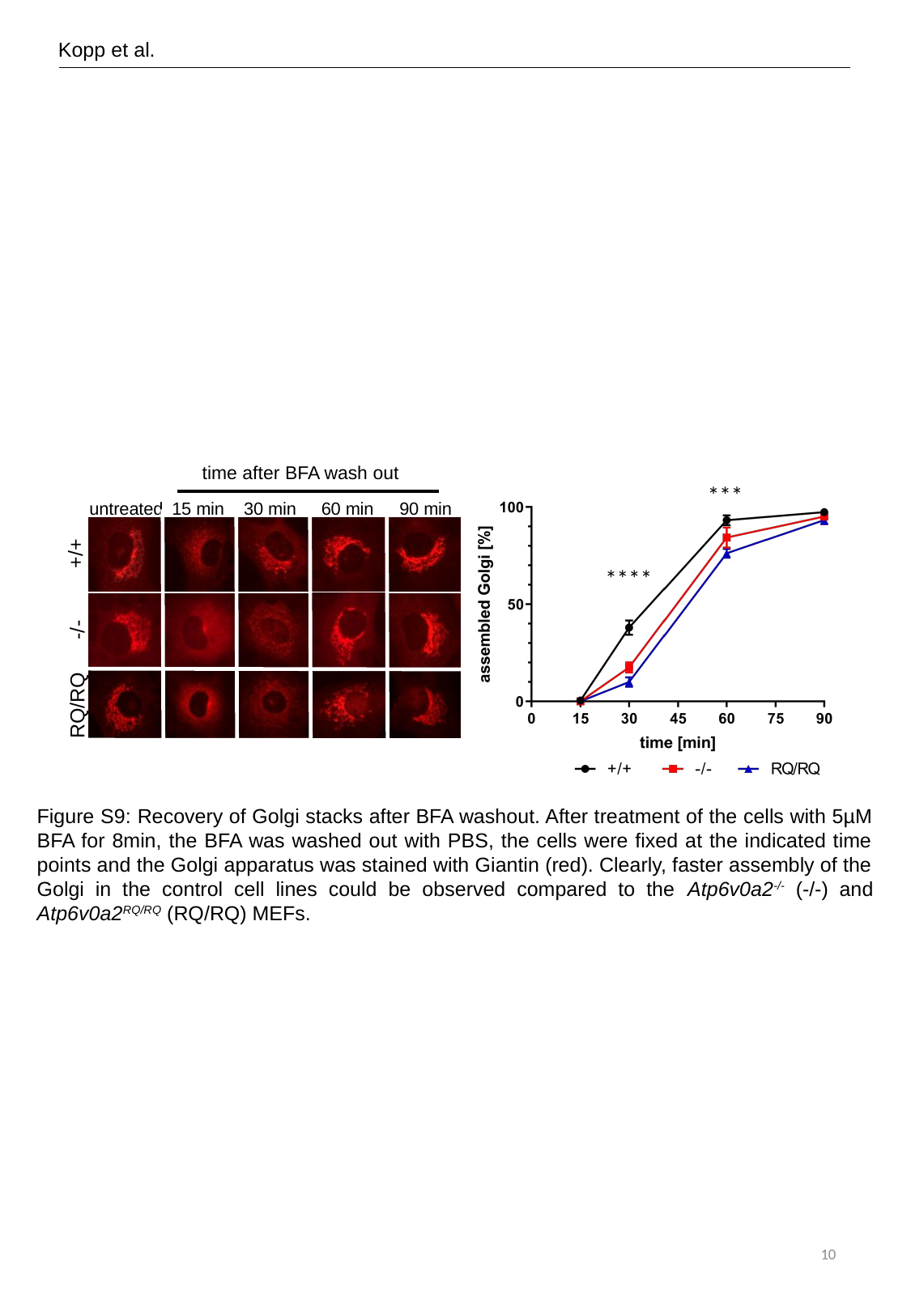

Kopp et al.
time after BFA wash out
***
untreated
15 min
30 min
60 min
90 min
+/+
****
-/-
RQ/RQ
Figure S9: Recovery of Golgi stacks after BFA washout. After treatment of the cells with 5µM BFA for 8min, the BFA was washed out with PBS, the cells were fixed at the indicated time points and the Golgi apparatus was stained with Giantin (red). Clearly, faster assembly of the Golgi in the control cell lines could be observed compared to the Atp6v0a2-/- (-/-) and Atp6v0a2RQ/RQ (RQ/RQ) MEFs.
10

## Slide 11
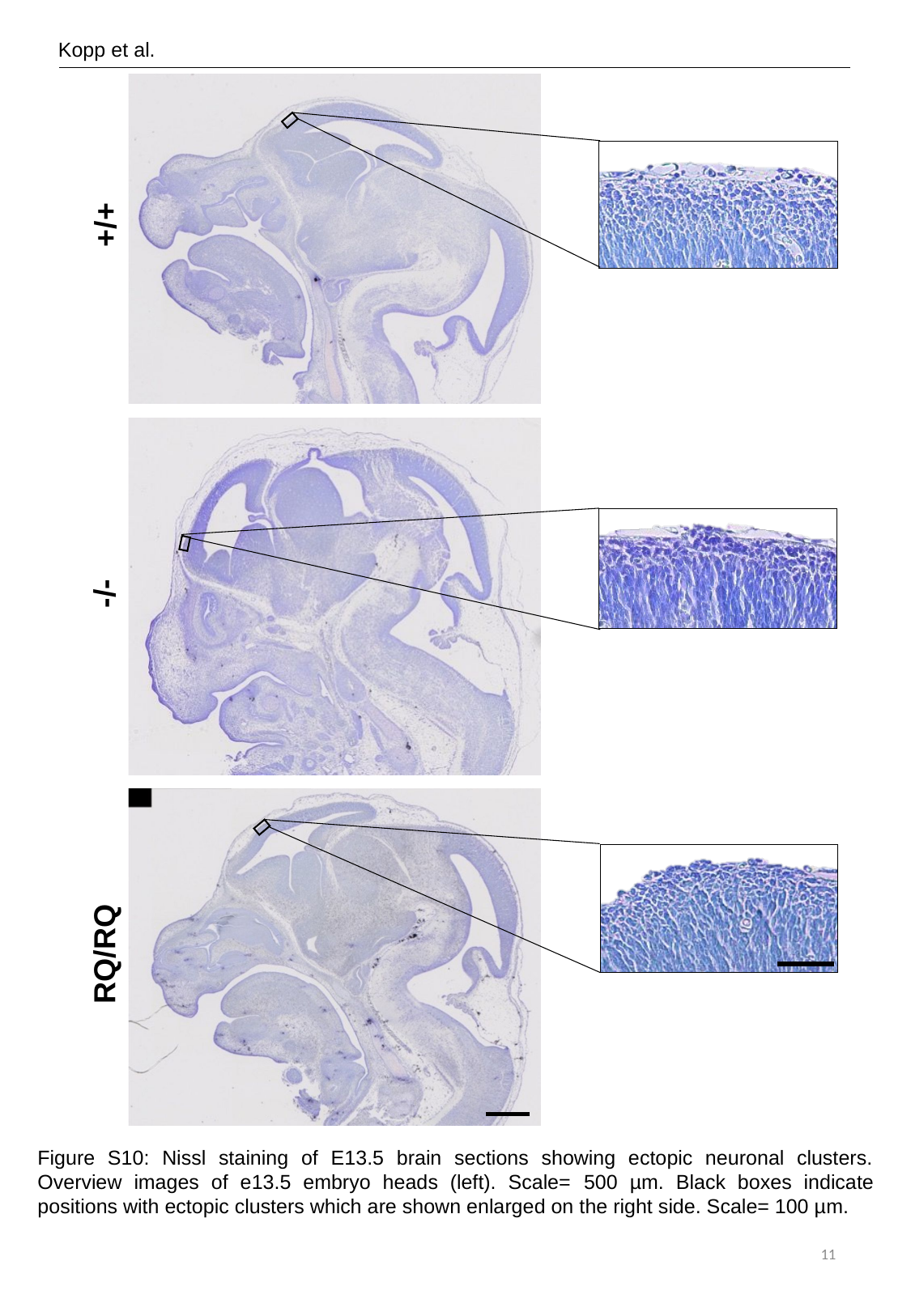

Kopp et al.
+/+
-/-
RQ/RQ
Figure S10: Nissl staining of E13.5 brain sections showing ectopic neuronal clusters. Overview images of e13.5 embryo heads (left). Scale= 500 µm. Black boxes indicate positions with ectopic clusters which are shown enlarged on the right side. Scale= 100 µm.
11

## Slide 12
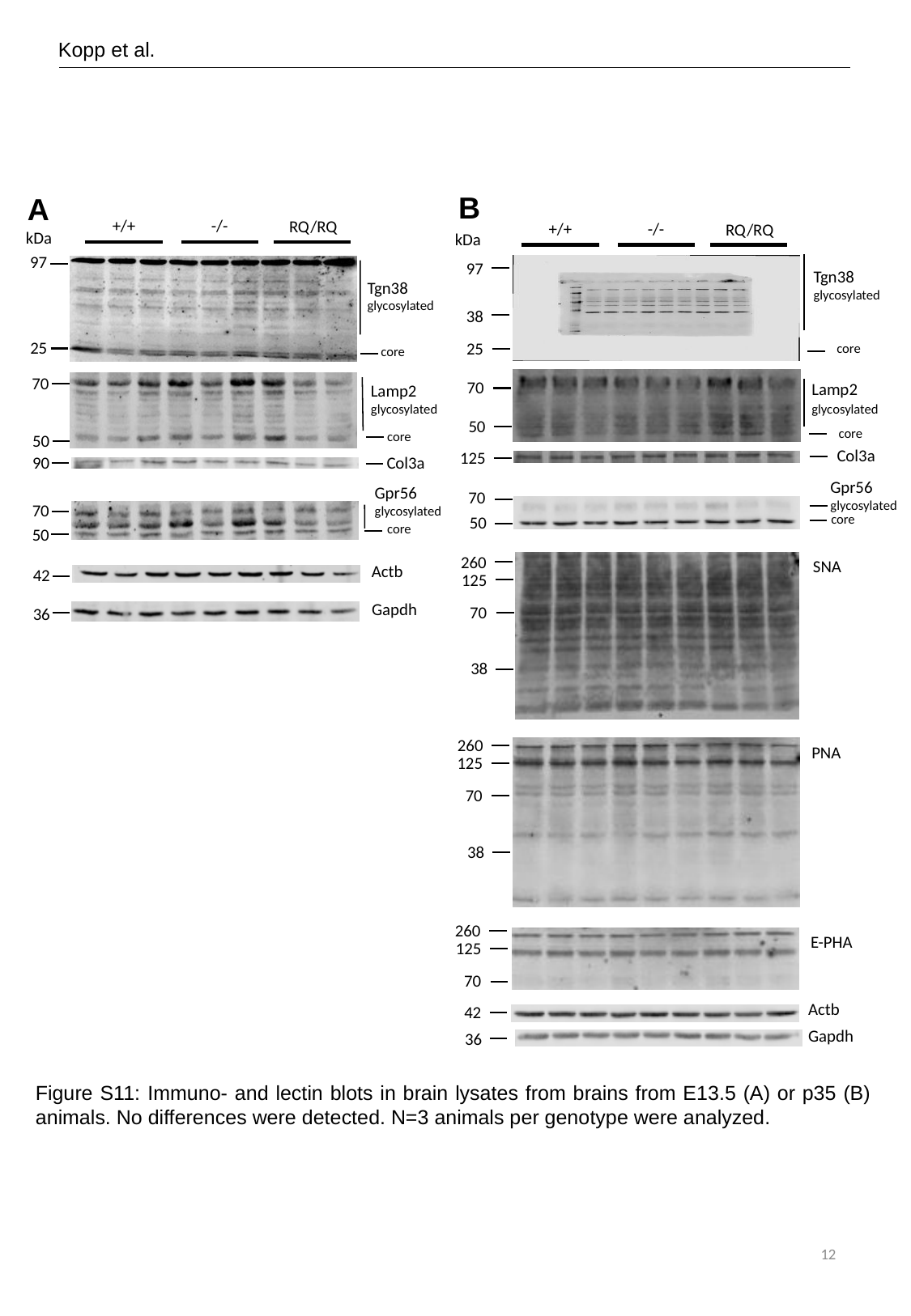

Kopp et al.
B
-/-
+/+
RQ/RQ
kDa
97
Tgn38
glycosylated
38
core
25
Lamp2
glycosylated
70
50
core
Col3a
125
Gpr56
glycosylated
core
70
50
260
SNA
125
70
38
260
PNA
125
70
38
260
E-PHA
125
70
Actb
42
Gapdh
36
A
-/-
+/+
RQ/RQ
kDa
97
Tgn38
glycosylated
25
core
70
Lamp2
glycosylated
core
50
Col3a
90
Gpr56
glycosylated
70
core
50
Actb
42
Gapdh
36
Figure S11: Immuno- and lectin blots in brain lysates from brains from E13.5 (A) or p35 (B) animals. No differences were detected. N=3 animals per genotype were analyzed.
12

## Slide 13
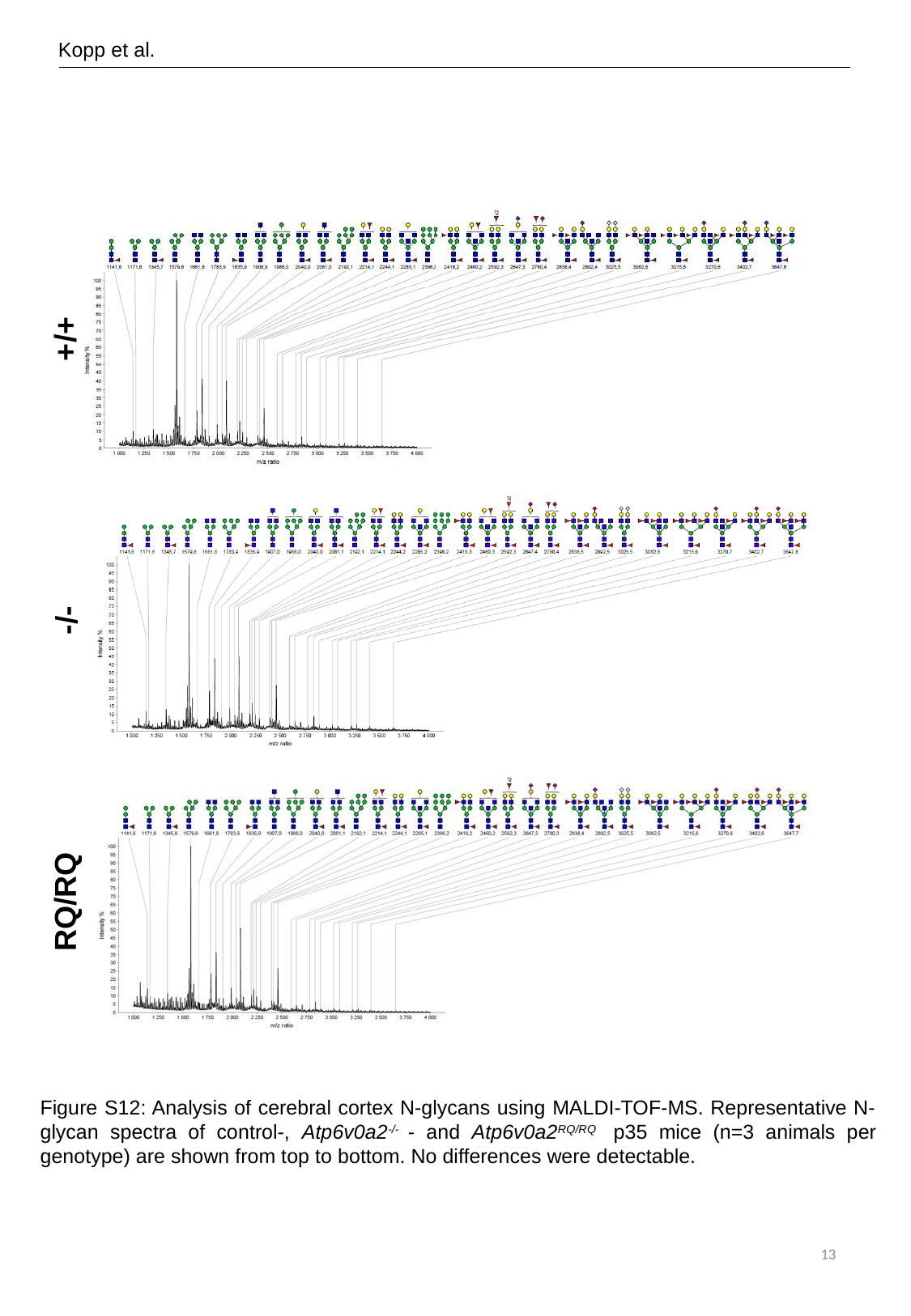

Kopp et al.
+/+
-/-
RQ/RQ
Figure S12: Analysis of cerebral cortex N-glycans using MALDI-TOF-MS. Representative N-glycan spectra of control-, Atp6v0a2-/- - and Atp6v0a2RQ/RQ  p35 mice (n=3 animals per genotype) are shown from top to bottom. No differences were detectable.
13

## Slide 14
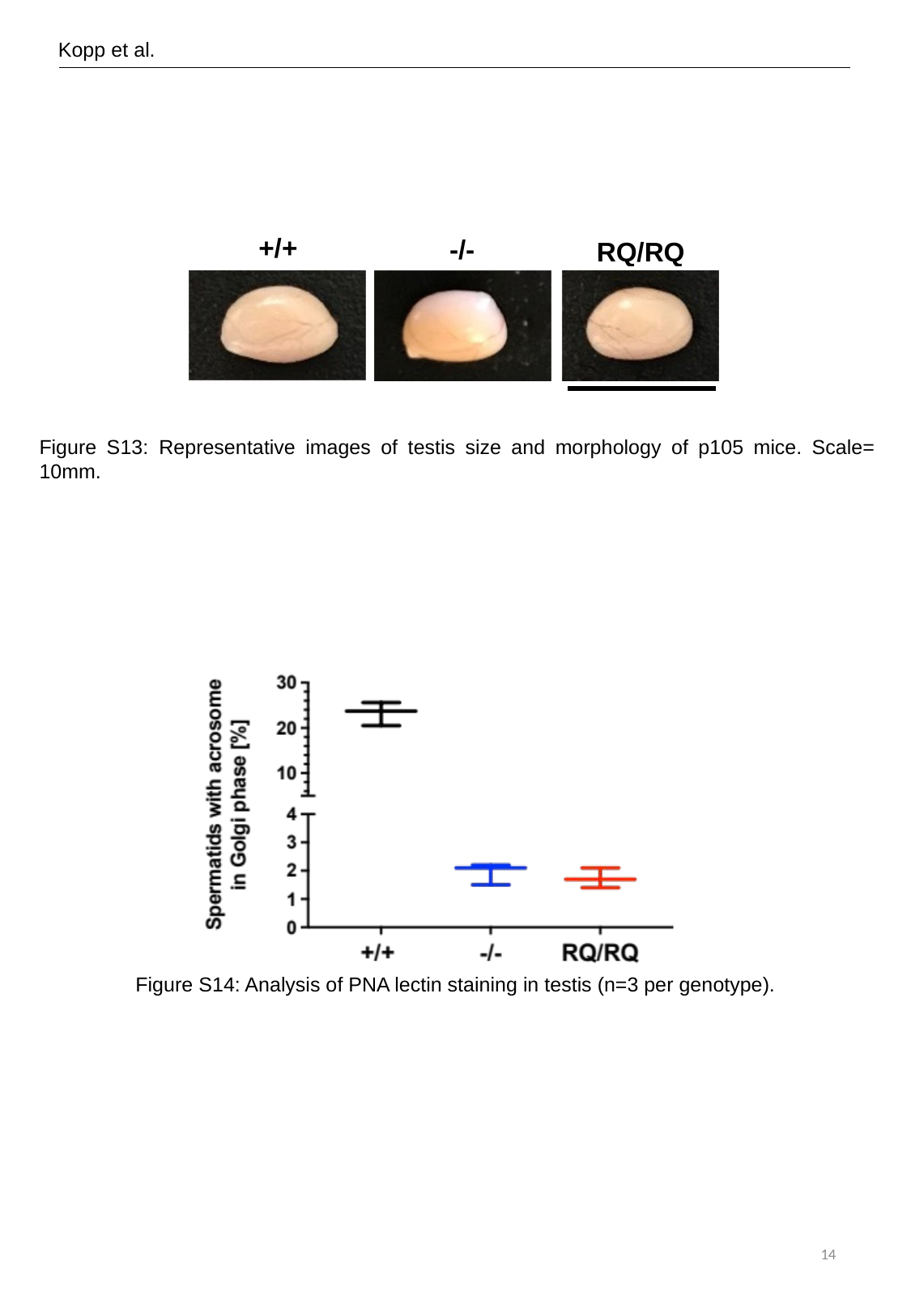

Kopp et al.
+/+
-/-
RQ/RQ
Figure S13: Representative images of testis size and morphology of p105 mice. Scale= 10mm.
Figure S14: Analysis of PNA lectin staining in testis (n=3 per genotype).
14

## Slide 15
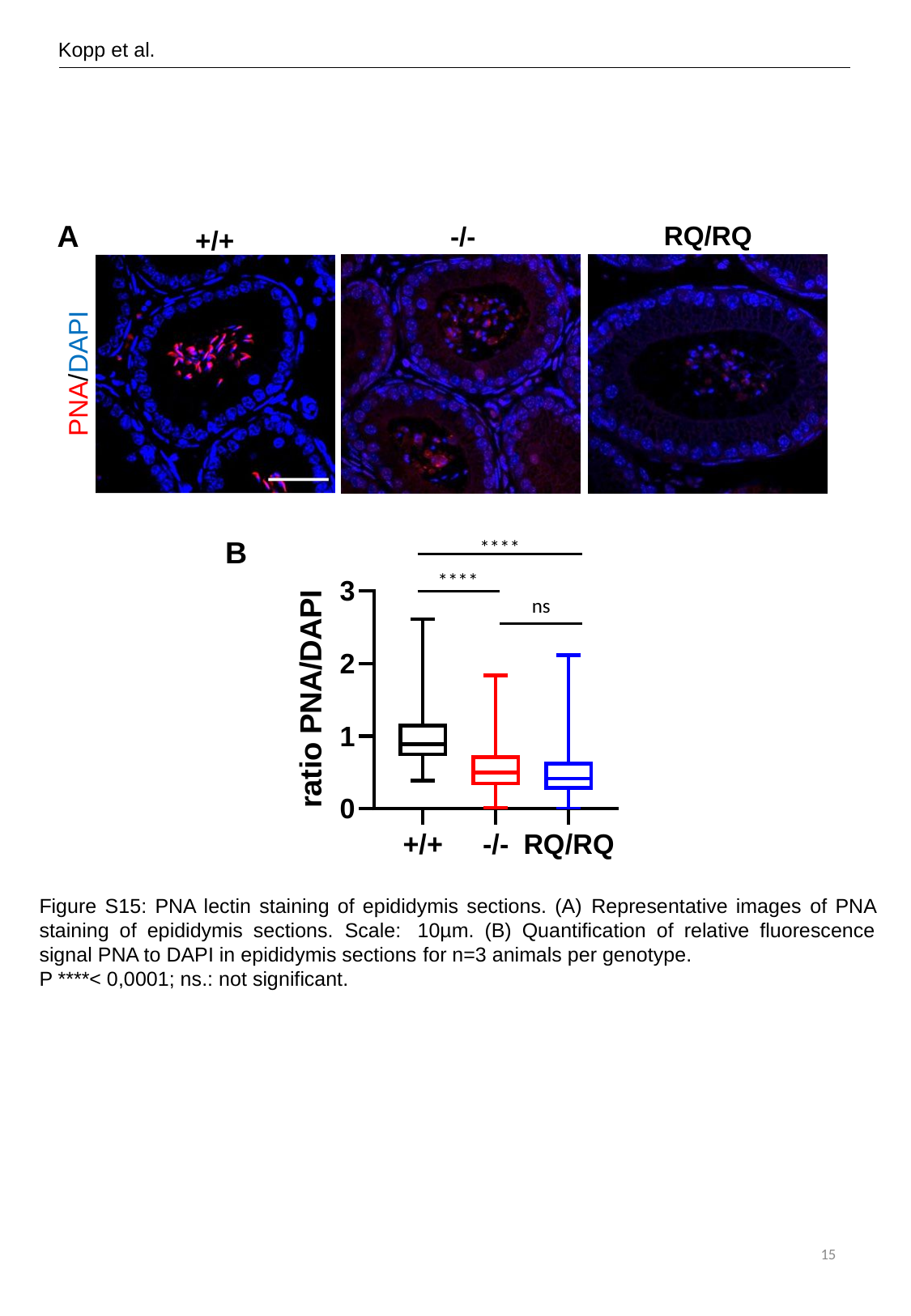

Kopp et al.
RQ/RQ
-/-
A
# +/+
PNA/DAPI
B
****
****
ns
Figure S15: PNA lectin staining of epididymis sections. (A) Representative images of PNA staining of epididymis sections. Scale:  10µm. (B) Quantification of relative fluorescence signal PNA to DAPI in epididymis sections for n=3 animals per genotype.
P ****< 0,0001; ns.: not significant.
15

## Slide 16
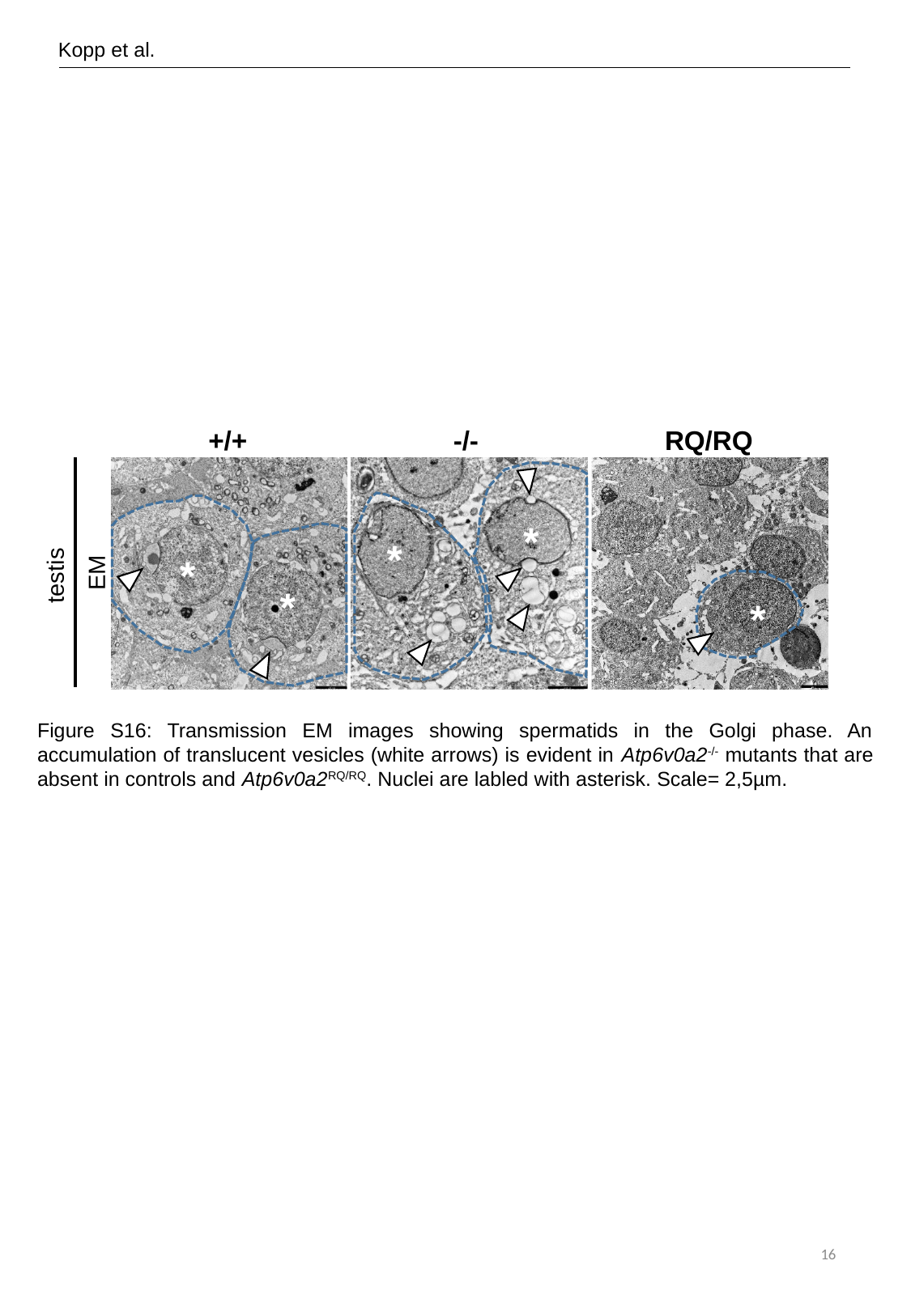

Kopp et al.
-/-
RQ/RQ
+/+
*
*
*
*
*
EM
testis
Figure S16: Transmission EM images showing spermatids in the Golgi phase. An accumulation of translucent vesicles (white arrows) is evident in Atp6v0a2-/- mutants that are absent in controls and Atp6v0a2RQ/RQ. Nuclei are labled with asterisk. Scale= 2,5µm.
16

## Slide 17
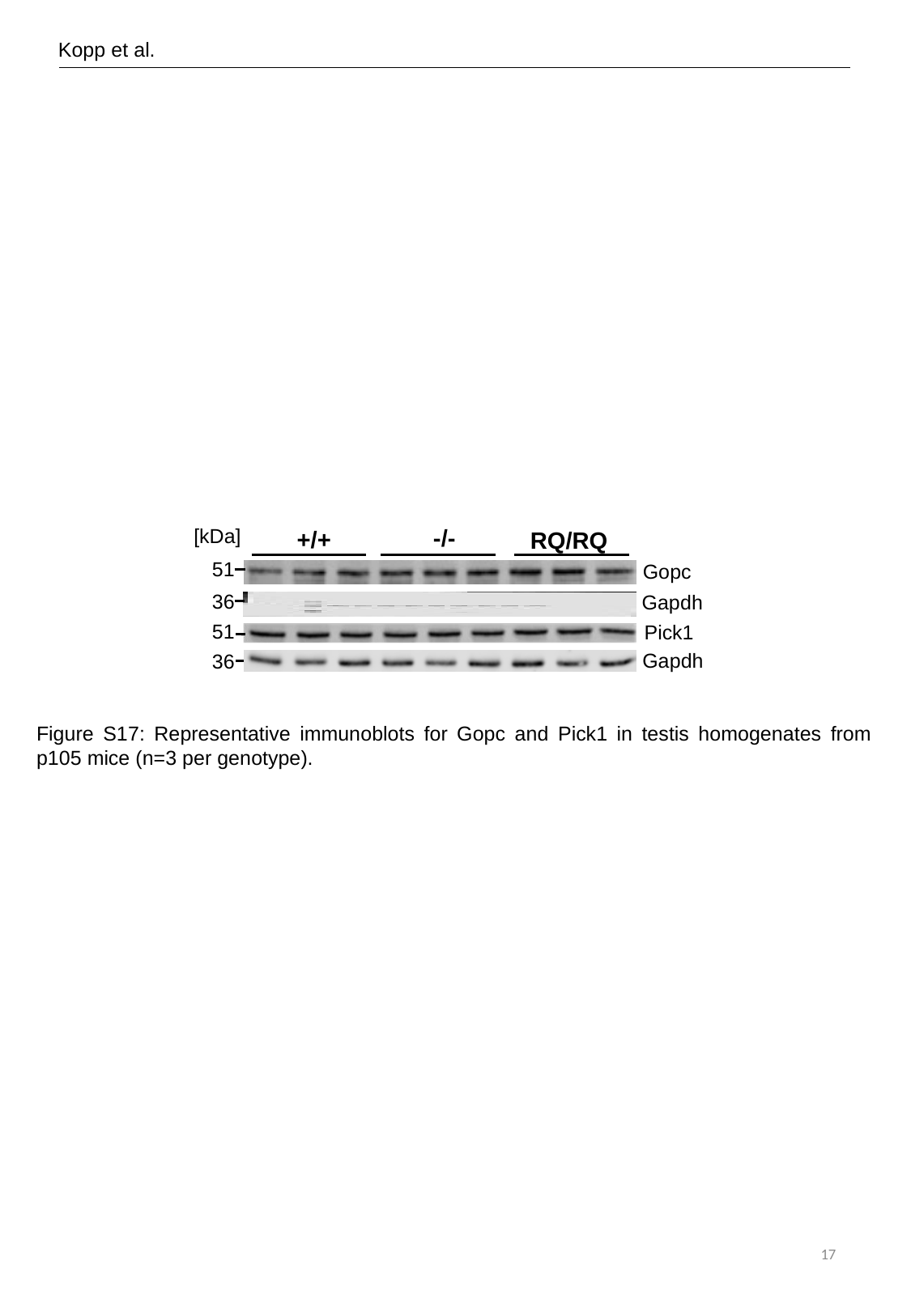

Kopp et al.
[kDa]
-/-
+/+
RQ/RQ
51
Gopc
36
Gapdh
51
Pick1
Gapdh
36
Figure S17: Representative immunoblots for Gopc and Pick1 in testis homogenates from p105 mice (n=3 per genotype).
17

## Slide 18
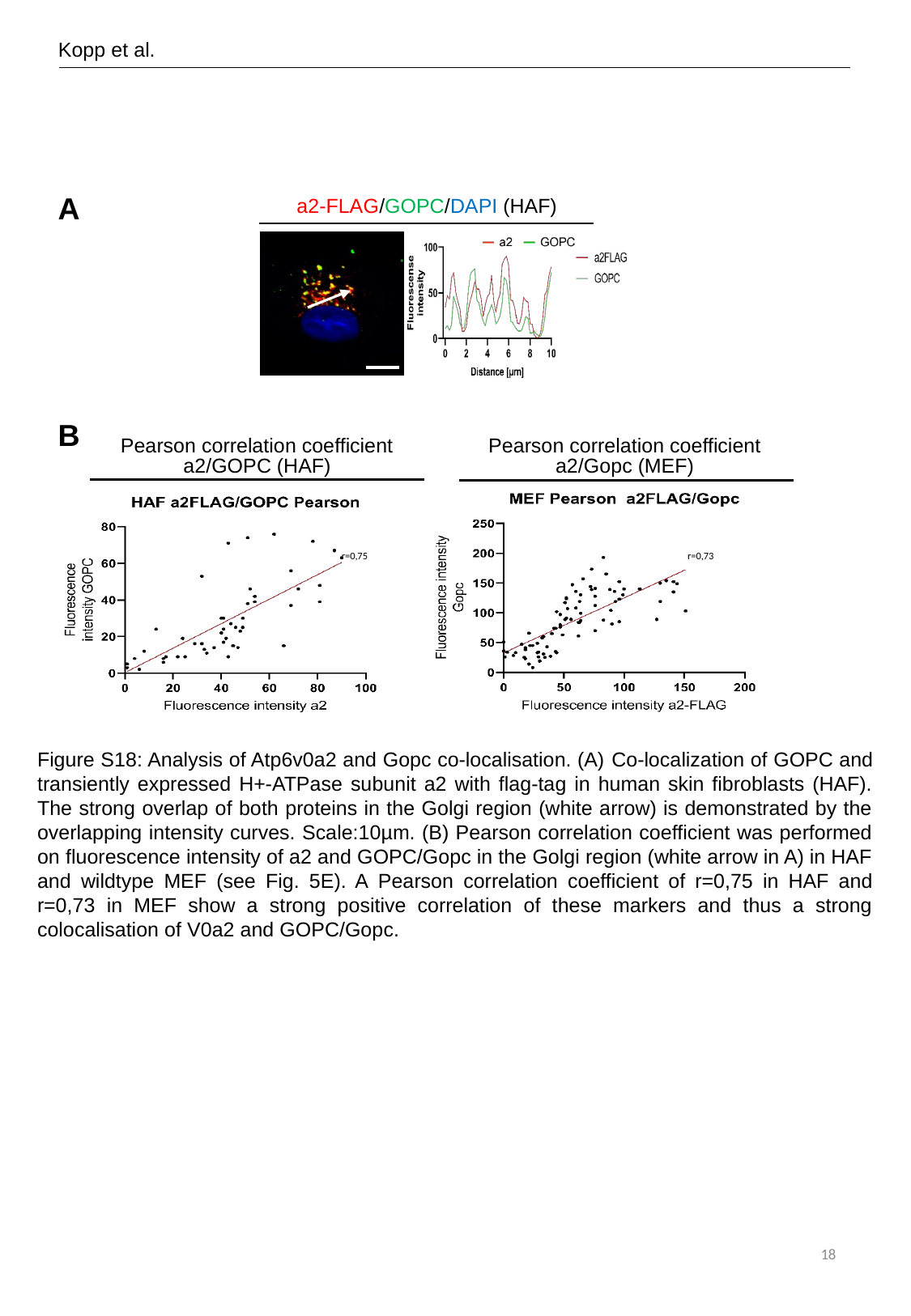

Kopp et al.
A
a2-FLAG/GOPC/DAPI (HAF)
B
Pearson correlation coefficient a2/Gopc (MEF)
Pearson correlation coefficient a2/GOPC (HAF)
r=0,75
r=0,73
Figure S18: Analysis of Atp6v0a2 and Gopc co-localisation. (A) Co-localization of GOPC and transiently expressed H+-ATPase subunit a2 with flag-tag in human skin fibroblasts (HAF). The strong overlap of both proteins in the Golgi region (white arrow) is demonstrated by the overlapping intensity curves. Scale:10µm. (B) Pearson correlation coefficient was performed on fluorescence intensity of a2 and GOPC/Gopc in the Golgi region (white arrow in A) in HAF and wildtype MEF (see Fig. 5E). A Pearson correlation coefficient of r=0,75 in HAF and r=0,73 in MEF show a strong positive correlation of these markers and thus a strong colocalisation of V0a2 and GOPC/Gopc.
18

## Slide 19
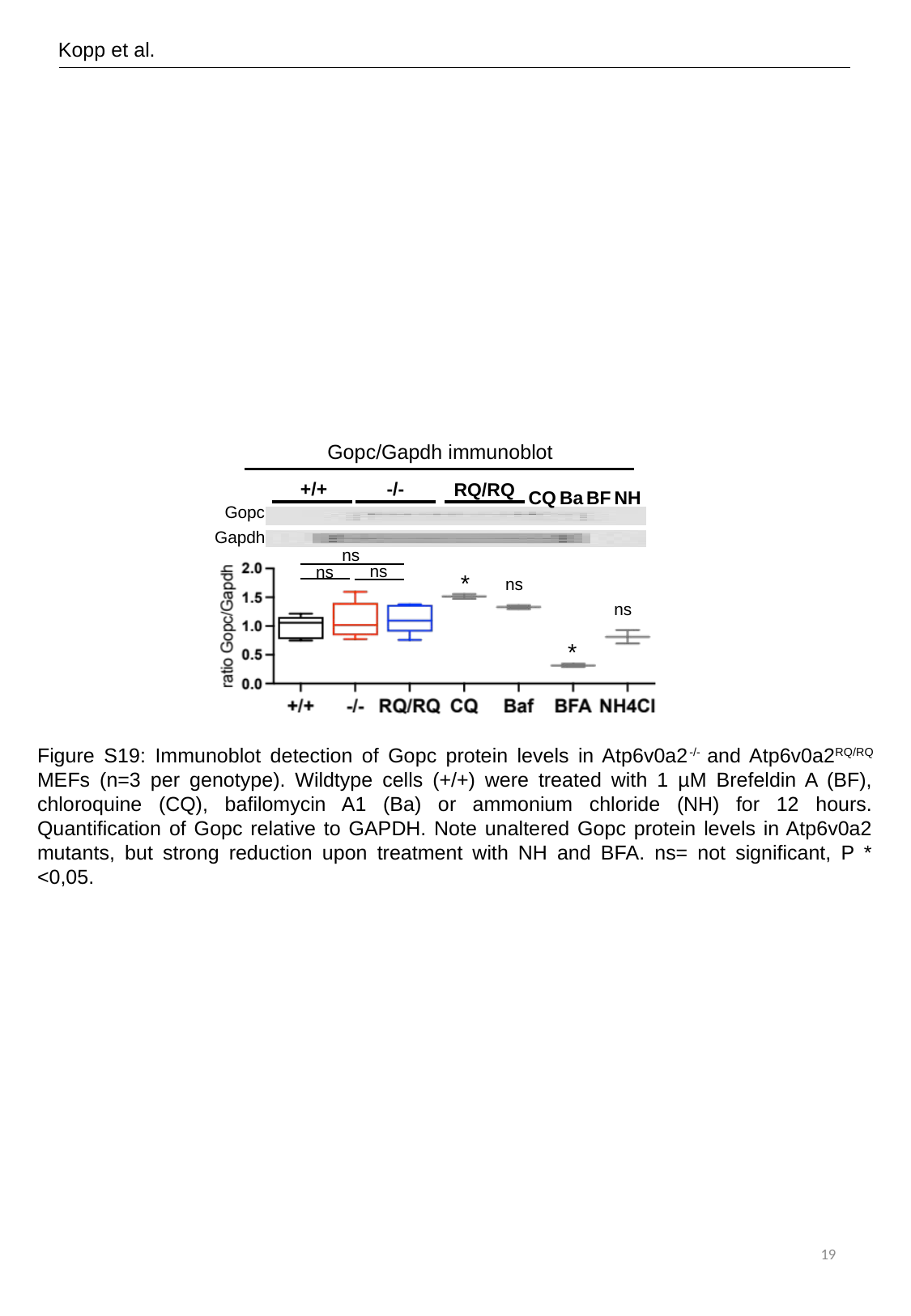

Kopp et al.
Gopc/Gapdh immunoblot
-/-
+/+
RQ/RQ
Ba
BF
NH
CQ
Gopc
Gapdh
ns
ns
ns
*
ns
ns
*
Figure S19: Immunoblot detection of Gopc protein levels in Atp6v0a2-/- and Atp6v0a2RQ/RQ MEFs (n=3 per genotype). Wildtype cells (+/+) were treated with 1 µM Brefeldin A (BF), chloroquine (CQ), bafilomycin A1 (Ba) or ammonium chloride (NH) for 12 hours. Quantification of Gopc relative to GAPDH. Note unaltered Gopc protein levels in Atp6v0a2 mutants, but strong reduction upon treatment with NH and BFA. ns= not significant, P * <0,05.
19
